# Genome‐wide analysis of adolescent psychotic‐like experiences shows genetic overlap with psychiatric disorders

**DOI:** 10.1002/ajmg.b.32630

**Published:** 2018-03-30

**Authors:** Oliver Pain, Frank Dudbridge, Alastair G. Cardno, Daniel Freeman, Yi Lu, Sebastian Lundstrom, Paul Lichtenstein, Angelica Ronald

**Affiliations:** ^1^ Department of Psychological Sciences Birkbeck, University of London London United Kingdom; ^2^ Department of Non‐Communicable Disease Epidemiology London School of Hygiene and Tropical Medicine London United Kingdom; ^3^ Academic Unit of Psychiatry and Behavioural Sciences University of Leeds Leeds United Kingdom; ^4^ Department of Psychiatry University of Oxford Oxford United Kingdom; ^5^ Department of Medical Epidemiology and Biostatistics Karolinska Institute Stockholm Sweden; ^6^ Centre for Ethics, Law and Mental Health (CELAM) University of Gothenburg Gothenburg Sweden; ^7^ Gillberg Neuropsychiatry Centre University of Gothenburg Gothenburg Sweden

**Keywords:** adolescence, ALSPAC, GWAS, psychotic‐like experiences, schizophrenia

## Abstract

This study aimed to test for overlap in genetic influences between psychotic‐like experience traits shown by adolescents in the community, and clinically‐recognized psychiatric disorders in adulthood, specifically schizophrenia, bipolar disorder, and major depression. The full spectra of psychotic‐like experience domains, both in terms of their severity and type (positive, cognitive, and negative), were assessed using self‐ and parent‐ratings in three European community samples aged 15–19 years (*Final N incl. siblings* = 6,297–10,098). A mega‐genome‐wide association study (mega‐GWAS) for each psychotic‐like experience domain was performed. Single nucleotide polymorphism (SNP)‐heritability of each psychotic‐like experience domain was estimated using genomic‐relatedness‐based restricted maximum‐likelihood (GREML) and linkage disequilibrium‐ (LD‐) score regression. Genetic overlap between specific psychotic‐like experience domains and schizophrenia, bipolar disorder, and major depression was assessed using polygenic risk score (PRS) and LD‐score regression. GREML returned SNP‐heritability estimates of 3–9% for psychotic‐like experience trait domains, with higher estimates for less skewed traits (Anhedonia, Cognitive Disorganization) than for more skewed traits (Paranoia and Hallucinations, Parent‐rated Negative Symptoms). Mega‐GWAS analysis identified one genome‐wide significant association for Anhedonia within *IDO2* but which did not replicate in an independent sample. PRS analysis revealed that the schizophrenia PRS significantly predicted all adolescent psychotic‐like experience trait domains (Paranoia and Hallucinations only in non‐zero scorers). The major depression PRS significantly predicted Anhedonia and Parent‐rated Negative Symptoms in adolescence. Psychotic‐like experiences during adolescence in the community show additive genetic effects and partly share genetic influences with clinically‐recognized psychiatric disorders, specifically schizophrenia and major depression.

## 1 INTRODUCTION

Psychotic‐like experiences, also referred to as psychotic experiences, are traits in the community that at the extreme resemble symptoms of psychotic disorders, such as schizophrenia. Based on principal component analyses, psychotic‐like experiences can be separated into replicable and specific domains, such as positive, cognitive, and negative domains (Ronald et al., 2014; Wigman et al., 2012). Psychotic‐like experiences are common in the general population, particularly during adolescence (Freeman, 2006; Ronald et al., 2014). Evidence suggests that psychotic‐like experiences are dimensional: they show varying degrees of severity and taxometric analyses support their continuous nature in adolescence (Ahmed, Buckley, & Mabe, 2012; Daneluzzo et al., 2009; Taylor, Freeman, & Ronald, 2016). Adolescence is just prior to a peak time of onset for several psychiatric disorders, particularly schizophrenia, bipolar disorder, and major depression (Laursen, Munk‐Olsen, Nordentoft, & Mortensen, 2007). Psychotic‐like experiences predict many types of psychiatric disorders and suicidal ideation with significant odds ratios of 1.3–5.6 (Cederlöf et al., 2016; Fisher et al., 2013; Kelleher et al., 2014, 2012; McGrath et al., 2016; Werbeloff et al., 2012; Zammit et al., 2013).

Twin studies report modest heritability of psychotic‐like experience domains with typically between a third and a half of the variance explained by genetic influences (Ericson, Tuvblad, Raine, Young‐Wolff, & Baker, 2011; Hay et al., 2001; Hur, Cherny, & Sham, 2012; Linney et al., 2003; Polanczyk et al., 2010; Wigman et al., 2011; Zavos et al., 2014). One study to date has reported single nucleotide polymorphism (SNP)‐heritability estimates ranging from 0 to 32%. These results suggest that common genetic variation plays a role in at least some types of adolescent psychotic‐like experiences (Sieradzka et al., 2015), but larger studies are needed to offer accurate estimates.

The one previous genome‐wide association study (GWAS) of adolescent psychotic‐like experiences assigned 3,483 individuals to high‐ or low‐scoring groups based on a measure of positive psychotic‐like experiences that combined paranoia, hallucinations, and delusions (Zammit et al., 2014). No locus achieved genome‐wide significance.

The Psychiatric Genomics Consortium 2 schizophrenia GWAS has been previously used twice to test for an association between genetic risk of schizophrenia and adolescent psychotic‐like experiences (these followed one study using Psychiatric Genomics Consortium 1 schizophrenia GWAS results; Zammit et al., 2014). The first of these studies used quantitative measures of individual psychotic‐like experiences in adolescence in a sample of *N* = 2,133–2,140 and reported no significant positive association between any of the psychotic‐like experience domains and schizophrenia genetic risk (Sieradzka et al., 2014). This study also reported no significant positive association with bipolar disorder genetic risk. A second study with *N* = 3,676–5,444 tested for an association between schizophrenia genetic risk and dichotomous outcomes on several scales: a combined positive psychotic‐like experiences scale (which included paranoia, hallucinations, delusions, and thought interference), a negative symptoms scale, a depressive disorder scale, and an anxiety disorder scale (Jones et al., 2016). This second study reported a significant positive association between schizophrenia genetic risk and both high negative symptoms and high anxiety disorder. There was no association with the positive psychotic‐like experiences or depressive disorder scales.

Schizotypal traits are closely related to psychotic‐like experiences. Schizotypal traits focus on differences in personality that reflect liability to psychotic disorders rather than the presentation of subclinical psychotic‐like experiences. Previous twin studies of schizotypal traits report modest heritability (Ericson et al., 2011; Lin et al., 2007), and studies are starting to explore the link between polygenic risk for schizophrenia and schizotypal traits (Hatzimanolis et al., 2017). In light of the differences in the constructs of schizotypy and psychotic‐like experiences, we focused our brief review on psychotic‐like experiences.

Our study aimed to test for overlap in genetic influences between specific psychotic‐like experience domains in adolescence and psychiatric disorders—specifically, schizophrenia, bipolar disorder, and major depression. Four psychotic‐like experience subscales were used, derived from principal component analysis: Paranoia and Hallucinations, Cognitive Disorganization, Anhedonia, and Parent‐rated Negative Symptoms. We also present the largest GWAS to date of adolescent psychotic‐like experiences using a community sample of 6,297–10,098 individuals (including siblings) from three European studies. As such our study also aimed to identify novel common genetic variants associated with specific psychotic‐like experience domains and to estimate their SNP‐heritability.

## 2 MATERIALS AND METHODS

### 2.1 Samples

Three European general population samples of comparable ages were included: Twins Early Development Study (TEDS) (Haworth, Davis, & Plomin, 2013) a community sample born in England and Wales (mean age 16.32 years); Avon Longitudinal Study of Parents and Children (ALSPAC) (Boyd et al., 2012) a cohort from the United Kingdom (mean age 16.76 years); Child and Adolescent Twin Study in Sweden (CATSS; Anckarsäter et al., 2011) a twin cohort in Sweden (mean age 18.31 years). Sample descriptions and ethical consent information are available in Supporting Information Note S1. Standard exclusion criteria for GWASs of behavioral and cognitive traits were used (Supporting Information Table S1) (Docherty et al., 2010).

### 2.2 Measures

We established comparable quantitative scales of psychotic‐like experience domains across samples. The subscales in the Specific Psychotic Experiences Questionnaire, used in TEDS, were the starting point (Ronald et al., 2014). The other two samples had used similar self‐report items that were mapped onto domains of paranoia, hallucinations, cognitive disorganization, anhedonia, and parent‐rated negative symptoms. The original measures available within each sample are listed in Supporting Information Note S2. The content of each psychotic‐like experience domain used within each sample is given in Supporting Information Tables S2–S5. Cronbach's alphas (measure of reliability) of each scale within each sample are listed in Table 1.

**Table 1:** Descriptive statistics for psychotic‐like experience domains in each sample

Specific PE	μ^a^	σ^b^	Range	Skew	α^c^	*N* d	*N* sibs^e^
TEDS							
Paranoia and hallucinations	0.43	0.51	0–3.90	2.03	0.73	2,994	827
Anhedonia	3.19	1.54	0–9.80	0.53	0.79	2,988	821
Cognitive disorganization	3.83	2.82	0–11.00	0.51	0.77	2,987	823
Parent‐rated negative symptoms	0.88	1.19	0–9.33	2.35	0.84	2,995	833
ALSPAC							
Paranoia and hallucinations	0.27	0.51	0–4.17	2.78	0.67	3,591	0
Anhedonia	1.42	1.19	0–7.00	1.09	0.73	3,591	0
Parent‐rated negative symptoms	1.57	1.11	0–7.00	0.61	0.61	4,019	0
CATSS							
Paranoia and hallucinations	0.20	0.43	0–5.00	3.92	0.73	2,080	849
Cognitive disorganization	1.95	0.98	0–5.00	0.35	0.79	3,310	1,335
Parent‐rated negative symptoms	0.57	0.95	0–8.50	2.64	0.75	3,084	1,395

*Note*. These figures are based on the individuals remaining after quality control and were used in all subsequent genetic analyses. ALSPAC = Avon Longitudinal Study of Parents and Children; CATSS = Child and Adolescent Twin Study in Sweden; TEDS = Twins Early Development Study.

a Mean.

b Standard deviation.

c Standardized Cronbach's alpha.

d Number of genotyped individuals after exclusions.

e Number of siblings pairs within *N*.

### 2.3 Phenotypic harmonization

Items were inspected to allow for harmonization of psychotic‐like experience domains across samples via a two‐stage process. First, two expert clinicians (AC and DF) matched items across samples that were capturing the same underlying construct (to ensure construct and content validity) based on their clinical knowledge and experience with psychotic‐like experience measures. Second, the matched items within each sample were analyzed via principal components analysis, a psychometric approach used to determine individual components underlying the items. This process was used to identify the presence of distinct psychotic‐like experience domains.

Individuals with >50% missingness for any psychotic‐like experience domain were excluded from all analyses. The remaining missing phenotypic data was imputed using multiple imputation in R (Buuren & Groothuis‐Oudshoorn, 2011). Imputation of item level data was carried out separately for each sample and psychotic‐like experience domain. Individual scores of the resulting psychotic‐like experience subscales were calculated using sum scores. To ensure the equal contribution of each item to the sum score, item response values were rescaled to values between 0 and 1. The phenotypic correlations between specific psychotic‐like experiences within each sample and in all samples combined are in Supporting Information Table S6. Sum scores for each psychotic‐like experience subscale were normalized using inverse rank‐based normalization (data ties ranked randomly) and then standardized. The median correlation between a given scale before and after normalization was 0.92 (Supporting Information Table S7), dependent on skew of the original scale. The normalized scores were then regressed against the following covariates: sex, age, age^2^, sex*age, sex*age^2^, study, and the top 8 principal components of ancestry. Principal components of ancestry were jointly calculated across all three samples using PLINK1.9 (https://www.cog-genomics.org/plink2) (Chang et al., 2015) based only on observed genetic variation.

### 2.4 Genotype imputation and quality control procedure

Genotypic data from each sample was imputed using the 1KG Phase 3 v5 to maximize genome‐wide overlap between samples. After imputation, stringent variant and individual level quality control thresholds were applied in all samples before converting to hard‐call genotypes (certainty threshold = 0.9) and merging the three data sets for combined analysis. For further information see Supporting Information Note S3. Information on DNA collection and genotyping is given in Supporting Information Note S4.

### 2.5 Estimation of SNP‐heritability

SNP‐heritability estimates were calculated within each sample and then meta‐analyzed to provide meta‐SNP‐heritability estimates. The meta‐analysis of SNP‐heritability estimates approach was taken to avoid a downward bias due to possible genetic heterogeneity between samples. Inverse variance weighted meta‐analysis was used. Secondary analysis of mega‐SNP‐heritability, estimated across samples simultaneously, was also performed to estimate the proportion of phenotypic variance that could be explained by the mean genetic effects across samples.

Two methods were used to estimate SNP‐heritability: genomic‐relatedness‐matrix restricted maximum‐likelihood (GREML) in genome‐wide complex trait analysis (GCTA), and linkage disequilibrium (LD)‐score regression. Related individuals were included in both meta‐ and mega‐GREML analyses (Zaitlen et al., 2013). LD‐score regression (Bulik‐Sullivan et al., 2015) was also performed within (meta‐) and across (mega‐) samples. The effective sample size was used in LD‐score regression analyses, thus matching the sample in the GWAS. Effective sample size was calculated as follows: (2*sample size)/(1 + correlation between siblings) (Minică, Boomsma, Vink, Dolan, 2014). There was no evidence of confounding from genome‐wide association results so the intercept was constrained to 1.

### 2.6 Mega‐GWAS

The three samples were combined to enable genome‐wide association mega‐analysis. Genome‐wide association analysis of all four psychotic‐like experience domains using related (i.e. monozygotic and dizygotic twin pairs) and unrelated individuals was performed in PLINK (http://pngu.mgh.harvard.edu/purcell/plink/) (Purcell et al., 2007). Additional covariance arises from related individuals; this was accounted for using the method of generalizable estimating equations (GEEs) (Minică et al., 2014; Minică, Dolan, Kampert, Boomsma, & Vink, 2015) in R specifying an exchangeable correlation matrix.

### 2.7 Replication analysis of rs149957215 association with Anhedonia

After the discovery sample had been prepared and analyzed for this project, an independent subsample of TEDS participants was genotyped. Of the newly genotyped individuals, 2,359 (incl. 635 MZ pairs) had reported on self‐rated Anhedonia. This independent TEDS sample was used as a replication sample for the validation of the genome‐wide significant association between rs149957215 and Anhedonia identified by the mega‐GWAS in this study. rs149957215 genotypes were imputed (MACH *r* ^2^ = 0.93) using the haplotype reference consortium data via the Sanger imputation server (McCarthy et al., 2016). The genotypic data was converted to hard‐call format (certainty threshold of 0.9) and analyzed in PLINK using the same GEE method to account for relatives. This replication analysis had a power of 0.86 to detect an association of the same magnitude (*r* ^2^ = 0.47%) at nominal significance.

### 2.8 Gene‐based association analysis

Two gene‐based association analyses were performed. The first aggregates SNP associations within specific gene regions using the MAGMA program (de Leeuw, Mooij, Heskes, & Posthuma, 2015). The second analysis used PrediXcan (Gamazon et al., 2015) to predict frontal cortex gene expression differences using genotypic data. Further details can be found in Supporting Information Note S5.

### 2.9 Genetic association between psychotic‐like experience domains and psychiatric disorders

Using the software PRSice (Euesden, Lewis, & O'Reilly, 2015), polygenic risk scores (PRSs) of schizophrenia, bipolar disorder, and major depression were calculated in the adolescent sample using the log of the odds ratios from the latest Psychiatric Genomics Consortium GWAS of schizophrenia (PGC2) (Ripke et al., 2014), bipolar disorder (PGC Bipolar Disorder Working Group, 2011), and major depression (Ripke et al., 2013). LD was controlled for using LD‐based clumping with an *r* ^2^‐cutoff of 0.1 within a 250‐kb window. For each individual, scores were generated using SNPs with the following *p*‐value thresholds (*p*T): 0.001, 0.01, 0.05, 0.1, 0.2, 0.3, 0.4, and 0.5. Linear regressions were performed in R and GEE was used to account for related individuals. Quantile plots were created to further examine the relationships. If there was evidence of a non‐linear relationship from the quantile plots, the non‐linear relationship was formalized by performing linear regression in a subset of individuals.

To estimate the genetic covariance between psychotic‐like experience domains and schizophrenia, bipolar disorder, and major depression, both LD‐score regression and additive variance explained and number of genetic effects method of estimation (AVENGEME) were used (Bulik‐Sullivan et al., 2015; Palla & Dudbridge, 2015). AVENGEME uses the results of PRS analyses across multiple significance thresholds to estimate the model parameters including the genetic covariance. AVENGEME estimates 95% confidence intervals using profile likelihood method. There was no evidence of confounding or sample overlap in the mega‐GWAS summary statistics, as such the heritability‐intercept was constrained to 1 and the genetic covariance intercept was set to 0 in LD‐score regression. To improve the accuracy of the estimates of genetic covariance derived from the AVENGEME analysis, the SNP‐heritability of liability for schizophrenia, bipolar disorder, and major depression were constrained to the LD‐score regression estimates of SNP‐heritability (see Supporting Information Table S8).

## 3 RESULTS

Table 1 shows the descriptive statistics of psychotic‐like experience domains split by sample. The relationship between each psychotic‐like experience domain with age and sex are available in Supporting Information Tables S9 and S10. After phenotypic harmonization and quality control, the final sample sizes (including siblings) for all subsequent analyses were 8,665 for Paranoia and Hallucinations, 6,579 for Anhedonia, 6,297 for Cognitive Disorganization, and 10,098 for Parent‐rated Negative Symptoms.

SNP‐heritability estimates from meta‐GREML and meta‐LD‐score regression were between 2.8–8.8% and 6.6–21.5%, respectively (Table 2). Results from secondary analysis of SNP‐heritability using the mega‐analysis approach are given in Supporting Information Table S11.

**Table 2:** SNP‐heritability estimates for psychotic‐like experience domains

	Meta‐GREML			Meta‐LDSC		
Specific PE	SNP‐*h* ^2^ a	*SE*	*p*	SNP‐*h* ^2^ a	*SE*	*p*
Paranoia and hallucinations	0.028	0.050	.293	0.066	0.068	.168
Anhedonia	0.088	0.055	.057	0.204	0.078	4.51 × 10^−3^
Cognitive disorganization	0.059	0.063	.176	0.215	0.085	5.79 × 10^−3^
Parent‐rated negative symptoms	0.059	0.045	.096	0.119	0.061	.026

a Phenotypic variance explained by tagged common genetic variation.

The mega‐GWAS identified no genome‐wide significant variation for the Paranoia and Hallucinations, Cognitive Disorganization, or Parent‐rated Negative Symptoms domains. The mega‐GWAS of Anhedonia identified one SNP (rs149957215) associated at genome‐wide significance (*p* = 3.76 × 10^−8^) within a gene called indoleamine 2,3‐dioxygenase 2 (*IDO2* **) (**Supporting Information Figure S1). This SNP was imputed in both samples included in the Anhedonia GWAS (TEDS and ALSPAC) with an average imputation quality (INFO) of 0.87 and minor allele frequency of 0.015. The minor allele frequency was similar in both TEDS (0.017) and ALSPAC (0.013). Due to limited LD with rs149957215, neighboring genetic variation showed no significant evidence of association. This association between rs149957215 and Anhedonia did not replicate in the independent TEDS replication sample, showing a non‐significant association (*p* = 0.81) in the opposite direction. Several loci achieved suggestive significance (*p* < 1 × 10^−5^) across the four psychotic‐like experience domains (Supporting Information Table S12 and Figures S2–S5). There was no evidence of confounding with lambdas of 0.99–1.01 and LD‐score regression intercept of 1.00 in all analyses (Supporting Information Figure S6).

Regional gene‐based tests identified no gene that was significantly associated with any psychotic‐like experience domain after Bonferroni correction for multiple testing. The top ten most associated genes for each psychotic‐like experience domain are listed in Supporting Information Table S13.

Analysis of predicted frontal cortex gene expression associated with psychotic‐like experience domains showed *HACD2* as significantly differentially expressed for Cognitive Disorganization (Bonferroni corrected *p* = 6.83 × 10^−4^). The top ten genes showing differential expression for each psychotic‐like experience domain are listed in Supporting Information Table S14.

**Figure 1:**
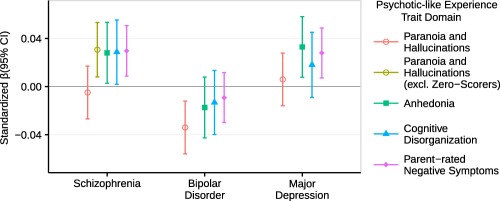
Polygenic risk scores for schizophrenia, bipolar disorder, and major depression predict adolescent psychotic‐like experience domains. This figure shows results for polygenic risk scores at the most predictive *p*‐value threshold for each trait. Error bars are 95% confidence intervals (95% CI) [Color figure can be viewed at http://wileyonlinelibrary.com]

The schizophrenia PRS significantly and positively predicted Anhedonia (*p* = .030 at *p*T = 0.10), Cognitive Disorganization (*p* = .035 at *p*T = 0.01) and Parent‐rated Negative Symptoms (*p* = 5.41 × 10^−3^ at *p*T = 0.05) (Table 3; Figure 1). The bipolar disorder PRS significantly and negatively predicted Paranoia and Hallucinations only (*p* = 2.47 × 10^−3^ at *p*T = 0.010) (Table 3; Figure 1). The major depression PRS significantly and positively predicted Anhedonia (*p* = .010 at *p*T 0.5) and Parent‐rated Negative Symptoms (*p* = 8.29 × 10^−3^ at *p*T = 0.001) (Table 3; Figure 1). Supporting Information Tables S15–S17 and Figures S7–S9 show the full results of these analyses. Logistic regression comparing PRSs in low and high psychotic‐like experience domain groups (defined as bottom and top 25% of raw psychotic‐like experience sum scores) were congruent with linear analyses (Supporting Information Table S18 and Figure S10).

**Table 3:** Schizophrenia, bipolar disorder, and major depression polygenic risk scores predicting psychotic‐like experience domains in adolescents

Specific PE	*β*	*SE*	*P*	*r* ^2^ (%)	*p*T^a^
Schizophrenia					
Paranoia and hallucinations	−0.005	0.011	.664	0.002	0.200
Paranoia and hallucinations excl. zero scorers	0.031	0.012	**7.90 × 10^−3^**	0.094	0.001
Anhedonia	0.028	0.013	**.030**	0.079	0.100
Cognitive disorganization	0.029	0.014	**.035**	0.083	0.010
Parent‐rated negative symptoms	0.030	0.011	**5.41 × 10^−3^**	0.088	0.050
Bipolar disorder					
Paranoia and hallucinations	−0.034	0.011	**2.47 × 10^−3^**	0.115	0.010
Anhedonia	−0.017	0.013	.178	0.030	0.100
Cognitive disorganization	−0.013	0.014	.333	0.017	0.001
Parent‐rated negative symptoms	−0.009	0.011	.388	0.008	0.500
Major depression					
Paranoia and hallucinations	0.006	0.011	.589	0.004	0.100
Anhedonia	0.033	0.013	**.010**	0.109	0.500
Cognitive disorganization	0.018	0.014	.189	0.033	0.050
Parent‐rated negative symptoms	0.028	0.011	**8.29 × 10^−3^**	0.078	0.001

This table shows results for polygenic risk scores at the most predictive *p*‐value threshold for each trait.

Statistically significant results are highlighted in bold.

a *p*‐value threshold used to select when calculating polygenic risk scores.

Quantile plots showing the mean PRS within subsets of the psychotic‐like experience distributions highlighted one non‐linear relationship between the schizophrenia PRS and Paranoia and Hallucinations, with the point of inflection at the median (Supporting Information Figure S11). The majority of individuals (81%) below the median had a raw score of zero. A single post‐hoc analysis was performed to formalize the presence of this non‐linear relationship. Post‐hoc removal of individuals with a raw Paranoia and Hallucinations score of zero led to the schizophrenia PRS positively predicting Paranoia and Hallucinations (*p* = 7.90 × 10^−3^ at *p*T = 0.001) (Table 3; Figure 1; Supporting Information Table S15). Logistic regression comparing low and high groups of non‐zero scoring individuals supported these findings (Supporting Information Table S18 and Figure S10).

Table 4 presents the AVENGEME estimates of genetic covariance, which were highly congruent with the PRS analysis results. AVENGEME pools evidence across *p*‐value thresholds tested from PRS analysis. As such, even when there is consistent but non‐significant evidence of association at individual *p*‐value thresholds, AVENGEME genetic covariance estimates can be significant. Consequently, there were two significant results that were not shown by the PRS analyses; between the Anhedonia psychotic‐like experience domain and bipolar disorder (genetic covariance = −0.022, 95% CI = −0.041 to −0.002), and the Cognitive Disorganization psychotic‐like experience domain and major depression (genetic covariance = 0.033, 95% CI = 0.005–0.062).

**Table 4:** Genetic covariance between each psychotic‐like experience domain and schizophrenia, bipolar disorder, and major depression

	AVENGEME			LDSC		
Specific PE	*cov*(G)^a^	Low 95% CI	High 95% CI	*cov*(G)^a^	*SE*	*p*
Schizophrenia						
Paranoia and hallucinations	−0.002	−0.013	0.008	−0.003	0.014	.844
Paranoia and hallucinations excl. zero scorers	**0.019**	0.011	0.029	NA	NA	NA
Anhedonia	**0.024**	0.013	0.038	0.021	0.016	.184
Cognitive disorganization	**0.025**	0.013	0.040	**0.054**	0.018	3.52 × 10^−3^
Parent‐rated negative symptoms	**0.025**	0.015	0.036	**0.047**	0.014	5.57 × 10^−4^
Bipolar disorder						
Paranoia and hallucinations	**−0.032**	−0.048	−0.018	**−0.045**	0.022	.039
Anhedonia	**−0.022**	−0.041	−0.002	0.008	0.028	.767
Cognitive disorganization	0.008	−0.013	0.029	0.021	0.028	.451
Parent‐rated negative symptoms	−0.010	−0.026	0.006	0.009	0.021	.669
Major depressive disorder						
Paranoia and hallucinations	0.004	−0.018	0.026	0.013	0.020	.523
Anhedonia	**0.065**	0.037	0.094	**0.061**	0.023	7.50 × 10^−3^
Cognitive disorganization	**0.033**	0.005	0.062	0.015	0.028	.594
Parent‐rated negative symptoms	**0.023**	0.010	0.042	0.031	0.020	.119

Statistically significant results are highlighted in bold.

a Genetic covariance.

Table 4 presents estimates of genetic covariance from LD‐score regression. LD‐score regression mirrored over half of the significant associations shown between the equivalent PRSs and psychotic‐like experience domains in Table 3. The genetic covariance between the schizophrenia PRS and non‐zero scorers on Paranoia and Hallucinations could not be estimated because the genome‐wide association analysis included zero scorers. Unlike for the equivalent results in Table 3, the genetic covariance between schizophrenia and anhedonia psychotic‐like experience domain, and between major depression and parent‐rated negative symptoms domain, were not significant.

## 4 DISCUSSION

A genetic relationship was identified between clinical schizophrenia and positive, cognitive, and negative psychotic‐like experience trait domains in adolescence. A higher genetic risk for schizophrenia significantly predicted adolescents having more cognitive disorganization, anhedonia, and parent‐rated negative symptoms, as well as more paranoia and hallucinations (this last finding was in the non‐zero scorers). Thus our findings suggest that psychotic‐like experience trait domains in adolescence comprise partly of the genetically‐influenced phenotypic manifestation of schizophrenia. Furthermore, higher genetic risk for major depression significantly predicted having more self‐rated anhedonia and parent‐rated negative symptoms as a teenager. Our results discredit the hypothesis that psychotic‐like experience trait domains in the general population are epiphenomena that do not share biological pathways with clinically‐recognized psychiatric disorders.

Genetic risk for schizophrenia and major depression in adulthood predicted 0.08–0.11% variance in psychotic‐like experience domains in adolescence. The effect sizes are likelydownward biased because of the conservative approaches taken here to align different samples and to handle skewed variables. Nevertheless, comparable effect sizes have been reported in equivalent studies on other phenotypes, for example, linking social communication traits to genetic liability for autism (St Pourcain et al., 2017). Large effect sizes were not expected. The upper limit of prediction is the degree to which PRSs for schizophrenia and major depression predict themselves (that is, their own phenotype) in an independent sample, which is ∼18.4% for schizophrenia (Ripke et al., 2014) and ∼0.6% for major depression (Ripke et al., 2013). It is known from epidemiological studies that the magnitude of phenotypic association between psychotic‐like experience domains and schizophrenia is modest (McGrath et al., 2016; Zammit et al., 2013) and far more people report psychotic‐like experiences in adolescence than develop disorders such as schizophrenia. Furthermore, there is considerable heterogeneity in schizophrenia and depression in terms of age of onset and symptom presentation. The associations were likely to be modest given their cross‐phenotype and cross‐age nature. The genetic association between psychiatric disorders and psychotic‐like experience traits in the community may increase with age or with longitudinal assessments.

A notable result was that paranoia and hallucinations during adolescence are associated with schizophrenia common genetic risk if individuals report at least some degree of paranoia or hallucinations, that is, the association was only present in the non‐zero scorers. Individuals reporting no paranoia and hallucinations can exist anywhere on the schizophrenia genetic liability spectrum. Previous studies did not find a genetic association between schizophrenia and positive psychotic‐like experiences (Jones et al., 2016; Sieradzka et al., 2014; Zammit et al., 2014). The use of quantitative traits and a large sample allowed the identification of this non‐linear effect here. An explanation for the non‐linear effect may lie in the variable age of onset of paranoia and hallucinations. Our study was focused on psychotic‐like experiences in mid to late adolescence. It is predicted that stronger positive associations between genetic risk for schizophrenia and positive psychotic‐like experiences will be found in samples assessed over a longer time frame, when anyone who is going to have paranoia and hallucinations has manifested them.

The significant and positive genetic association between a self‐rated anhedonia trait measure in adolescence and major depression in adulthood concurs with a previous report showing that subclinical depressive symptoms (including anhedonia) phenotypically predict major depressive episodes in adulthood (Pine, Cohen, Cohen, & Brook, 1999). Anhedonia is present as a symptom of both schizophrenia and depression in psychiatric diagnoses, and our research shows that as a trait dimension in adolescence it shares common genetic underpinnings with both schizophrenia and depression.

The significant negative association between paranoia and hallucinations and bipolar disorder genetic risk also deserves discussion. Previous research reported the presence of paranoia, hallucinations, and delusions prior to the onset of bipolar disorder (McGrath et al., 2016). However, our results suggest that genetic influences on paranoia and hallucinations as traits during mid‐adolescence are negatively associated with known common genetic risk associated with diagnosed bipolar disorder. The common genetic relationship between adolescent paranoia and hallucinations and bipolar disorder requires replication in an independent sample. Further insight into this relationship will also be gained as more powerful polygenic predictors for bipolar disorder become available.

Although in many cases the SNP‐heritability estimates were not significantly non‐zero, the point estimates of SNP‐heritability indicate that common genetic variation influences psychotic‐like experience domains during adolescence. This concurs with results from twin studies reporting significant twin heritability estimates (Zavos et al., 2014).

The SNP that achieved genome‐wide significance in the mega‐GWAS for anhedonia lies within the protein‐coding gene *IDO2*. IDO2 is a key enzyme in the regulation of the kynurenine pathway, which upon stimulation by proinflammatory cytokines, converts tryptophan into kynurenine. It has been reported that increased metabolism of tryptophan to kynurenine is associated with increased depressive symptoms via the increased production of cytotoxic kynurenine metabolites (Dantzer, O'Connor, Lawson, & Kelley, 2011; Myint et al., 2007; Wichers et al., 2005). In fact, a previous study has reported a significant correlation between kynurenine production and anhedonia in an adolescent sample (Gabbay, Ely, Babb, & Liebes, 2012). These previous studies suggest the association between *IDO2* and anhedonia is plausible. However, this association should be interpreted with caution as rs149957215 was imputed in all three samples, has a low minor allele frequency of 0.013, and appears to be uncorrelated with surrounding common genetic variation. Furthermore, this association failed to replicate in a sufficiently powered independent sample.

Some methodological details deserve consideration. A key strength of the study is the derivation of psychometrically‐sound quantitative individual psychotic‐like experience domains: These were derived using principal component analysis and have content validity. It is noted that our terminology of psychotic‐like experiences, like others (Alemany et al., 2013; Stefanis et al., 2002; Wigman et al., 2011), includes positive, cognitive, and negative psychotic‐like experiences, for the reason that at the extreme these experiences are characteristic of clinical psychotic disorders. Greater power was achieved compared to past research through combining independent samples. At the same time, slight variations in measure items across samples impacts the amount of phenotypic variance that can be explained by common genetic variation. The correlation between specific psychotic‐like experiences were typically consistent within each sample, however the relationship between Paranoia and Hallucinations and other psychotic‐like experiences did vary to some degree (Supporting Information Table S6). For the more skewed psychotic‐like experience domains with larger numbers of tied individuals, the process of randomly ranking tied individuals during normalization was essential but will have introduced noise and thus downward biased SNP‐heritability estimates. This study used multiple PRS selection thresholds (referred to as *p*Ts), however the *p*‐values for each selection threshold result have not been corrected for multiple testing. Due to the high correlation between PRS selection thresholds, even when considering all possible selection thresholds, it has been estimated that a significance threshold of *p* = .001 is appropriate (Euesden et al., 2015). This threshold would be overly conservative in our study as we only tested a limited number of thresholds. Genetic covariance estimates obtained by AVENGEME (Table 4) take all selection thresholds into account simultaneously, and therefore need no adjustment for multiple testing. We note that the significance of the PRS associations is supported by the confidence intervals of the AVENEMGE genetic covariance estimates. This study has identified significant common genetic covariance between several psychotic‐like experiences and psychiatric disorders. It remains possible that the genetic covariance estimates could be partly explained by the presence of adolescents with diagnosed relatives. If an adolescent has a relative with schizophrenia for example, the adolescent may have increased psychotic‐like experiences due to their shared environment with the relative. However, twin and adoption studies of schizophrenia and psychotic‐like experiences do not support this mode of transmission. Finally, each psychotic‐like experience domain was viewed as a single phenotype of interest in its own right. As such, this study was treated as a series of self‐contained individual trait studies, rather than as a single exploratory study across traits. Therefore we did not formally correct for multiple testing of traits, similar to other studies of this nature (Cross‐Disorder Group of the Psychiatric Genomics Consortium, 2013; Wellcome Trust Case Control Consortium, 2007). Note however that nearly all of our associations would remain significant after correction for testing four PE domains, while the association of rs149957215 with Anhedonia (*p* = 3.76×10^−8^) did not replicate.

Collectively, these findings reveal novel evidence for some shared common genetic etiology between psychotic‐like experience domains in mid to late adolescence and clinically‐recognized psychiatric disorders in adulthood. Evidence is accruing that psychotic‐like experiences manifested prior to adulthood form part of a wider phenotype or prodrome related to psychiatric disorders such as schizophrenia and depression. This study joins the existing evidence that individuals with a family history of schizophrenia score higher on psychotic‐like experience scales (Jeppesen et al., 2014; Zavos et al., 2014), psychotic‐like experiences are associated with the same environmental risk factors as schizophrenia (Linscott & Van Os, 2013), psychotic‐like experiences are on a phenotypic and etiological continuum across the severity continuum (Taylor et al., 2016; Zavos et al., 2014), and psychotic‐like experiences predict later psychiatric disorders (Cederlöf et al., 2016; Fisher et al., 2013; Kelleher, Cederlöf, & Lichtenstein, 2014; Kelleher et al., 2012; Werbeloff et al., 2012; McGrath et al., 2016; Zammit et al., 2013). The next step is to consider how psychotic‐like experiences can be harnessed in a practical sense as a (small effect size) red flag for risk in early intervention and prevention strategies. Similar to family history, psychotic‐like experiences might be a useful heuristic even if the majority of individuals with psychotic‐like experiences will remain unaffected by psychiatric disorders.

## CONFLICT OF INTEREST

The authors declared that they have no conflict of interest.

## Supporting information

Additional Supporting Information may be found online in the supporting information tab for this article.

Supporting Information
