# Supplementary material for: Genome‐wide analysis of adolescent psychotic‐like experiences shows genetic overlap with psychiatric disorders
Source: Am J Med Genet B Neuropsychiatr Genet. 2018 Mar 30;177(4):416–25. doi: 10.1002/ajmg.b.32630 (PMC6001485; doi:10.1002/ajmg.b.32630)
Supplement: Supplementary file 1 — Supporting Information [file AJMG-177-416-s001.docx]

**Genome-wide analysis of adolescent psychotic-like experiences shows genetic overlap with psychiatric disorders**

**Supplementary material**

**Supplementary Note 1.** Samples.

**Supplementary Note 2.** Measures.

**Supplementary Note 3.** Genotype imputation and harmonization.

**Supplementary Note 4.** DNA Collection and Genotyping.

**Supplementary Note 5.** Gene-based association analysis.

**Supplementary Table 1.** Exclusion variables.

**Supplementary Table 2.** Paranoia and Hallucinations measures.

**Supplementary Table 3.** Anhedonia measures.

**Supplementary Table 4.** Cognitive Disorganization measures.

**Supplementary Table 5.** Parent-rated Negative Symptoms measures.

**Supplementary Table 6.** Phenotypic correlation between specific adolescent psychotic-like experiences within and across samples.

**Supplementary Table 7.** Correlation between raw sum scores and scores after normalization.

**Supplementary Table 8.** Parameters used in AVENGEME analysis.

**Supplementary Table 9.** Mean sex differences for untransformed psychotic-like experience sum scores.

**Supplementary Table 10.** Pearson correlation between untransformed psychotic-like experience sum scores and age.

**Supplementary Table 11.** Mega-SNP-heritability estimates from GREML and LD-score regression.

**Supplementary Table 12.** Suggestive loci from mega-GWASs.

**Supplementary Table 13.** Summary of MAGMA results.

**Supplementary Table 14.** Summary of PrediXcan results.

**Supplementary Table 15.** Schizophrenia polygenic risk score predicting psychotic-like experience domains.

**Supplementary Table 16.** Bipolar disorder polygenic risk score predicting psychotic-like experience domains.

**Supplementary Table 17.** Major depression polygenic risk score predicting psychotic-like experience domains.

**Supplementary Table 18.** Comparison of polygenic risk scores among low and high psychotic-like experience domain groups using logistic regression.

**Supplementary Figure 1.** LocusZoom plot of genome-wide significant association with Anhedonia.

**Supplementary Figure 2.** Manhattan plot of Paranoia and Hallucinations mega-GWAS.

**Supplementary Figure 3.** Manhattan plot of Anhedonia mega-GWAS.

**Supplementary Figure 4.** Manhattan plot of Cognitive Disorganization mega-GWAS.

**Supplementary Figure 5.** Manhattan plot of Parent-rated Negative Symptoms mega-GWAS.

**Supplementary Figure 6.** Quantile-quantile plots of mega-GWAS results.

**Supplementary Figure 7.** Schizophrenia polygenic risk score predicting psychotic-like experience domains in adolescence.

**Supplementary Figure 8.** Bipolar disorder polygenic risk score predicting psychotic-like experience domains in adolescence.

**Supplementary Figure 9.** Major depression polygenic risk score predicting psychotic-like experience domains in adolescence.

**Supplementary Figure 10.** Schizophrenia, bipolar disorder, and major depression polygenic risk score mean differences between low- and high-scoring psychotic-like experience domain groups.

**Supplementary Figure 11.** Relationship between Paranoia and Hallucinations and schizophrenia polygenic risk score using local polynomial regression.

### **Supplementary Note 1.** Samples

**TEDS** [Haworth et al., 2013] – This sample recruited twins born in England and Wales between 1994 and 1996 and assessed these individuals longitudinally. TEDS originally recruited 13,488 families, who responded with a written consent form. The Institute of Psychiatry ethics committee approved TEDS (ref: 05/Q0706/228). Zygosity of twins was determined either using parental questionnaires (accuracy >95% [Haworth et al., 2013]) or DNA.

**ALSPAC** [Boyd et al., 2012] – This sample includes 14,775 children born to pregnant residents of the former Avon region in South West England who had an expected date of delivery between the 1^st^ of April 1991, and the 31^st^ of December 1992. Informed consent has been received from all participants within this study. Ethical approval for this study was obtained from the ALSPAC Law and Ethics Committee and the Local Research Ethics Committees. Please note that the study website contains details of all the data that is available through a fully searchable data dictionary (http://www.bristol.ac.uk/alspac/researchers/access/).

**CATSS** [Anckarsäter et al., 2011] – The CATSS sample includes all twins born in Sweden since the 1^st^ July 1992. Currently, data are available for ~5,350 twins at age 18 years from the CATSS-18 subset. All participants are informed of the information being collected and are repeatedly given the opportunity to withdraw. The study has ethical approval from the Karolinska Institute Ethical Review Board. Zygosity of twins was determined either using an intra-pair similarity algorithm (accuracy 98% [Magnusson et al., 2013]) or DNA.

### **Supplementary Note 2.** Measures

**TEDS** [Haworth et al., 2013] – The Specific Psychotic Experiences Questionnaire (SPEQ) [Ronald et al., 2014] consists of six subscales: self-rated paranoia, hallucinations, grandiosity, cognitive disorganization, anhedonia, and parent-rated negative symptoms.

**ALSPAC** [Boyd et al., 2012] – Individuals completed the Psychotic Like Experiences Questionnaire (PLIKS-Q) [Zammit et al., 2011]. The full instrument contains 11 questions asking about paranoia, hallucinations, delusions, and experiences of thought interference. The PLIKS-Q was included as a part of the ‘Life of 16+ Teenager’ questionnaire, which assessed a broader range of behaviours. Additional items that mirrored items from the SPEQ came from the Moods and Feelings questionnaire (MFQ) and the Strengths and Difficulties Questionnaire (SDQ) and parent-reported items in the ‘Your Son/Daughter 16+ Years On’ questionnaire.

**CATSS** [Anckarsäter et al., 2011] – Individuals completed the Adolescent Psychotic-like Symptom Screener (APSS) [Kelleher et al., 2011]. APSS assesses positive psychotic-like experiences (e.g. paranoia, hallucinations, and delusions). Additional items that mirrored items from the SPEQ came from the Child Mania Rating Scale (CMRS), the Adult ADHD Self-Report Scale (ASRS), and the Centre of Epidemiologic Studies Depression Scale (CES-D), as well as the parent-report Adult Behaviour Checklist (ABCL), and the Autism – Tics, ADHD and other Comorbidities Inventory (A-TAC).

The *Measures* section of the main text describes how items and scales were harmonized across samples. Supplementary Tables 2-5 list the items used.

### **Supplementary Note 3.** Genotype Imputation and Harmonization

Genotype imputation and harmonization was performed using only autosomes. The reference panel chosen for imputation was the 1KG Phase 3 version 5 dataset. Stringent quality control and strand alignment (ambiguous SNPs excluded) was performed prior to imputation. The data was phased using ShapeIt V2 and subsequently imputed in 5Mb sections using Impute2 [Howie et al., 2011, 2009]. In all cases the accuracy of imputation was between .90 and .99. SNPs with poor info scores were removed (INFO < .3). For ease of subsequent analysis, the dosage levels of imputed SNPs were converted to ‘best-guess’ format using a threshold of >.9 with the intention of following up associations of interest accounting for imputation probabilities.

The ‘best-guess’ genotype data from each sample were merged per chromosome using PLINK1.9. A light SNP-missingness threshold (>20%) was applied to remove SNPs that were neither imputed nor observed in the samples. A second round of stringent SNP- and individual-level QC was then applied: SNP missingness > 2%, individual missingness >5%, minor allele frequency (MAF) < 1%, Hardy-Weinberg equilibrium *p* > 1x10-6. After QC 4,487,870 common variants were captured in each of the samples

### **Supplementary Note 4.** DNA Collection and Genotyping

**TEDS:** DNA was extracted using buccal cheek swabs. Genotyping was performed using Affymetrix GeneChip 6.0 SNP genotyping platform at the Affymetrix Santa Clara, California, USA, as part of the TEDS Wellcome Trust Case Control Consortium 2 (WTCCC2) study of reading and mathematical abilities. Samples were excluded based on one or more of the following parameters: low call rate or heterozygosity outliers, atypical population ancestry, sample duplication or relatedness to other sample members, unusual hybridization intensity, gender mismatches, and having less than 90% of genotypes called identically on the genome-wide array and Sequenom panel. This resulted in 3,152 unrelated individuals being successfully genotyped consisting of 1 446 males and 1,706 females. A number of these individuals were from monozygotic (MZ) twin pairs enabling us to impute the genotype of their siblings based on the assumption that MZ twins are genetically identical. Given that both siblings of these genotyped MZ twin pairs provided phenotypic information, they were both included in subsequent analyses, accounting for family structure.

**ALSPAC:** DNA was extracted from umbilical cord blood. Genotyping was performed using the Illumina HumanHap550 quad genome-wide SNP genotyping platform by 23andMe subcontracting the Wellcome Trust Sanger Institute, Cambridge, UK and the Laboratory Corporation of America, Burlington NC, USA. Samples were excluded based on the following criteria: incorrect gender assignments, heterozygosity outliers, low call rate, cryptic relatedness and being of non-European ancestry as detected by a multidimensional scaling analysis seeded with HapMap 2 individuals. Subsequently, 8 365 unrelated individuals survived quality control consisting of 4,285 males and 4,080 females.

**CATSS:** DNA was extracted from saliva. Genotyping was performed using the Illumina Infinium PsychArray-24 BeadChip and carried out by SNP&SEQ Technologies in Uppsala, Sweden. Samples were excluded based on one or more of the following parameters: Low call rate, excess heterozygosity, sample duplication, erroneous within family relatedness, cryptic relatedness, gender mismatches, being non-European as detect by PCA seeded by the 1KG EUR (1000 genomes European) reference. This resulted in 17,898 individuals successfully genotyped. This number includes dizygotic (DZ) twin pairs. As in TEDS, the genotypes of MZ siblings were imputed if their co-twin was genotyped. If both siblings of these genotyped twin pairs provided phenotypic information, they were both included in subsequent analyses, while accounting for family structure.

### **Supplementary Note 5.** Gene-based association analysis

First, an approach was used that aggregates the association of SNPs within specified gene regions using software called MAGMA[de Leeuw et al., 2015]. MAGMA[de Leeuw et al., 2015] calculates gene-level association statistics using the results of each individual psychotic-like experience mega-GWAS. Genetic variants were assigned to genes based on the NCBI 37.3 build with a 10kb annotation window used around genes, resulting in 17,226 genes being tested. LD was calculated using the combined TEDS, ALSPAC, and CATSS sample. *p*-values were Bonferroni corrected to account for multiple testing.

Second, PrediXcan[Gamazon et al., 2015] was used which tests for an association between a given trait and predicted gene-expression. To estimate genetically-regulated gene-expression levels in individuals based on common genetic variation, it uses tissue-specific additive gene-expression prediction models that have been trained using reference transcriptomic datasets such as the GTeX (Genotype-Tissue Expression, http://www.gtexportal.org/home/) project. Here, the Frontal Cortex prediction model was used to estimate genetically regulated frontal cortex gene-expression levels in all individuals. Linear regression was used to test for an association between predicted gene-expression levels and individual PEs, using GEE to control for related individuals. After removal of genes with expression levels showing no variance, the total number of expression levels was 2,769. *p*-values were Bonferroni corrected to account for multiple testing.

Supplementary Table 1. Exclusion variables applied in each sample. These exclusion criteria are standard practice for genome-wide association studies of behavioral and cognitive traits [Docherty et al., 2010].

|  | **TEDS** | **ALSPAC** | **CATSS** |
| --- | --- | --- | --- |
| **Exclusion criteria** | - Unknown zygosity - Unknown sex at age 16 - Low birth weight - Short gestational age - Maternal drinking during pregnancy - Long stay in hospital after birth - Diagnosis of autism - Cerebral palsy - Genetic, chromosomal or inherited disorders - Brain damage or disorders affecting the brain - Severely deaf - Developmental delay - Complete blindness - Death of either twin | - Unknown sex - Low birth weight - Short gestational age - Maternal drinking during pregnancy - Long period in hospital after birth - Diagnosis of autism - Cerebral palsy - Severely deaf - Developmental delay | - Unknown zygosity - Unknown sex - Low birth weight - Birth trauma - Diagnosis of autism - Cerebral palsy - Chromosomal abnormalities - Brain damage - Deafness - Complete blindness |

Supplementary Table 2. Items for Paranoia and Hallucinations measures in each sample.

| ***TEDS*** |  |
| --- | --- |
|  |  |
| **Leading statement:** | How often have you thought… |
| **Response options:** | 0 = Not at all, 1 = Rarely, 2 = Once a month, 3 = Once a week, 4 = Several times a week, 5 = Daily |
|  |  |
| **Item 1:** | I might be being observed or followed? |
| **Item 2:** | I can detect coded messages about me in the press/TV/internet? |
| **Item 3:** | People might be conspiring against me? |
| **Item 4:** | Hear noises or sounds when there is nothing about to explain them? |
| **Item 5:** | Hear sounds or music that people near you don't hear? |
| **Item 6:** | Hear voices commenting on what you're thinking or doing? |
| **Item 7:** | See things that other people cannot? |
| **Item 8:** | See shapes, lights, or colours even though there is nothing really there? |
|  |  |
| Note: | In order to keep the same ratio of items assessing hallucinations to paranoia the same across samples, items 4,5 and 6 were averaged to create a composite auditory hallucinations item, and items 7 and 8 were average to create a composite visual hallucinations item. |
|  |  |
| ***ALSPAC*** |  |
|  |  |
| **Part A response options:** | 0 = No, never, 1 = Yes, maybe, 2 = Yes, definitely |
| **Part B response options:** | 0 = Not at all, 1 = Once or twice, 2 = Less than once a month, 3 = More than once a month, 4 = Nearly every day |
|  |  |
| **Item 1a:** | Some people believe that other people can read their thoughts. Have other people ever read your thoughts? |
| **Item 1b:** | How often have other people read your thoughts since your 15th birthday? |
| **Item 2a:** | Have you ever thought you were being followed or spied on? |
| **Item 2b:** | How often has this happened since your 15th birthday? |
| **Item 3a:** | Have you ever believed that you were being sent special messages though the television or the radio, or that a programme had been arranged just for you alone? |
| **Item 3b:** | How often has this happened since your 15th birthday? |
| **Item 4a:** | Have you ever seen something or someone that other people could not see? |
| **Item 4b:** | How often have you seen something or someone that other people could not see since your 15th birthday? |
| **Item 5a:** | Have you ever heard voices that other people couldn't hear |
| **Item 5b:** | How often have you heard voices that other people couldn't hear since your 15th birthday? |
|  |  |
| Note: | The responses to Part A and Part B were summed for each item. |
|  |  |
| ***CATSS*** |  |
|  |  |
| **Leading statement:** | Have you ever… |
| **Response options:** | 0 = Never or rarely, 1 = Sometimes, 2 = Often, 3 = Very Often |
|  |  |
| **Item 1:** | Thought you were being followed or spied on? |
| **Item 2:** | Thought you were being sent special messages through the television? |
| **Item 3:** | Thought other people could read your thoughts? |
| **Item 4:** | Seen things other people cannot see? |
| **Item 5:** | Heard voices that nobody else can hear? |

Supplementary Table 3. Items for Anhedonia measures in each sample.

| ***TEDS*** |  |
| --- | --- |
|  |  |
| **Response options:** | 0 = Very false for me, 1 = Moderately false for me, 2 = Slightly false for me, 3 = Slightly true for me, 4 = Moderately true for me, 6 = Very true for me |
|  |  |
| **Item 1:** | When something exciting is coming up in my life, I really look forward to it. (R) |
| **Item 2:** | When I'm on my way to an amusement park, I can hardly wait to ride the rollercoasters. (R) |
| **Item 3:** | When it think about eating my favourite food, I can almost taste how good it is. (R) |
| **Item 4:** | I don’t look forward to things like eating out at restaurants. |
| **Item 5:** | I get so excited the night before a major holiday I can hardly sleep. (R) |
| **Item 6:** | When I think of something tasty, like chocolate biscuit, I have to have one. (R) |
| **Item 7:** | Looking forward to a pleasurable experience is in itself pleasurable. (R) |
| **Item 8:** | I look forward to a lot of things in my life. (R) |
| **Item 9:** | When ordering something off a menu, I imagine how good it will taste. (R) |
| **Item 10:** | When I hear about a new movie starring my favourite actor, I can't wait to see it. (R) |
|  |  |
| ***ALSPAC*** |  |
|  |  |
| **Response options:** | 0 = No, never, 1 = Yes, sometimes, 2 = Yes, often, 3 = Yes, nearly always |
|  |  |
| **Item 1:** | Have you felt that you experience few or no emotions at important events, such as on your birthday? |
| **Item 2:** | Have you felt that you are lacking 'get up and go'? |
| **Item 3:** | Have you felt that you have only a few hobbies or interests? |
|  |  |
| **Leading statement:** | In the past two weeks… |
| **Response options:** | 0 = Not true, 1 = Sometimes true, 2 = True |
|  |  |
| **Item 4:** | I have been having fun. (R) |
| **Item 5:** | I didn't enjoy anything at all. |
| **Item 6:** | I felt so tired that I just sat around and did nothing. |
| **Item 7:** | I have had a good time. (R) |

Note. R = Reversed item.

Supplementary Table 4. Items for Cognitive Disorganization measures in each sample.

| ***TEDS*** |  |
| --- | --- |
|  |  |
| **Response options:** | 0 = Yes, 1 = No |
|  |  |
| **Item 1:** | Are you easily confused if too much happens at the same time? |
| **Item 2:** | Do you frequently have difficulty in starting to do things? |
| **Item 3:** | Are you a person whose mood goes up and down easily? |
| **Item 4:** | Do you dread going into a room by yourself where other people have already gathered and are talking? |
| **Item 5:** | Do you find it difficult to keep interested in the same thing for a long time? |
| **Item 6:** | Do you find it difficult in controlling your thoughts? |
| **Item 7:** | Are you easily distracted from work by daydreams? |
| **Item 8:** | Do you ever feel that your speech is difficult to understand because the words are all mixed up and don't make sense? |
| **Item 9:** | Are you easily distracted when you read or talk to someone? |
| **Item 10:** | Is it hard for you to make decisions? |
| **Item 11:** | When in a crowded room, do you often have difficulty in following a conversation? |
|  |  |
| ***CATSS*** |  |
|  |  |
| **Response options:** | 0 = Never, 1 = Rarely, 2 = Sometimes, 3 = Often, 4 = Very often |
|  |  |
| **Item 1:** | How often do you have trouble wrapping up the fine details of a project, once the challenging parts have been done? |
| **Item 2:** | When you have a task that requires a lot of thought, how often do you avoid or delay getting started? |
| **Item 3:** | How often do you have difficulty keeping your attention when you are doing boring or repetitive work? |
| **Item 4:** | How often do you have difficulty concentrating on what people say to you, even when they are speaking to you directly? |
| **Item 5:** | How often are you distracted by activity or noise around you? |

Supplementary Table 5. Items for Parent-rated Negative Symptoms measures in each sample.

| ***TEDS*** |  |
| --- | --- |
|  |  |
| **Leading statement:** | My child… |
| **Response options:** | 0 = Not at all true, 1 = Somewhat true, 2 = Mainly true, 3 = Definitely true |
|  |  |
| **Item 1:** | Usually gives brief, one word replies to questions, even if encouraged to say more. |
| **Item 2:** | Often does not have much to say for him/herself. |
| **Item 3:** | Has few or no friends. |
| **Item 4:** | Is often inattentive and appears distracted. |
| **Item 5:** | Often does not pay attention when being spoken to. |
| **Item 6:** | Often sits around for a long time doing nothing. |
| **Item 7:** | Has a lack of energy and motivation. |
| **Item 8:** | Has very few interests or hobbies. |
| **Item 9:** | Often fails to smile or laugh at things others would find funny. |
| **Item 10:** | Seems emotionally "flat", for example, rarely changes the emotions he/she shows. |
|  |  |
| ***ALSPAC*** |  |
|  |  |
| **Response options:** | 0 = Often, 1 = Sometimes, 2 = Hardly ever, 3 = Never |
|  |  |
| **Item 1:** | How often does he/she tell you about things that happen at school/college/work? |
| **Item 2:** | How often does he/she tell you about things that happen while he's/she's been out? |
|  |  |
| **Response options:** | 0 = No, 1 = Yes |
|  |  |
| **Item 3:** | Thinking back over the last month, has she been feeling tired or felt she had no energy? |
|  |  |
| **Leading statement:** | In the past 6 months… |
| **Response options:** | 0 = Not true, 1 = Somewhat true, 2 = Certainly true, NA = Don't know |
|  |  |
| **Item 4:** | He/She did not respond when told to do something. |
| **Item 5:** | He/She has at least one good friend. (R) |
| **Item 6:** | He/She is easily distracted, his/her concentration wanders. |
| **Item 7:** | He/She sees tasks through to the end, he/she has good attention span. |
|  |  |
| ***CATSS*** |  |
|  |  |
| **Leading statement:** | How accurate are the following statements for your child in the past six months? |
| **Response options:** | 0 = Not true, 1 = Somewhat true, 2 = Very or often true |
|  |  |
| **Item 1:** | Refuses to talk |
| **Item 2:** | Secretive, keeps things to self |
| **Item 3:** | Has trouble making or keeping friends |
| **Item 4:** | Withdrawn, doesn't get involved with others |
| **Item 5:** | Fails to finish things he/she should do |
| **Item 6:** | Can’t concentrate, can’t pay attention for long |
| **Item 7:** | Underactive, slow moving, or lacks energy |
| **Item 8:** | Feels tired without good reason |
| **Item 9:** | Stares blankly |
|  |  |
| **Response options:** | 0 = No, 1 = Yes, to a certain degree, 2 = Yes |
|  |  |
| **Item 10:** | Does the twin have difficulties expressing emotions and reactions with facial gestures, prosody, or body language? |

Note. R = Reversed item.

Supplementary Table 6. Phenotypic correlation between specific adolescent psychotic-like experiences within each sample and in all samples combined.

| ***TEDS, ALSPAC and CATSS*** |  |  |  |  |
| --- | --- | --- | --- | --- |
|  | **1** | **2** | **3** | **4** |
| ***1) Paranoia and Hallucinations*** | 1 | - | - | - |
| ***2) Anhedonia*** | 0.20 | 1 | - | - |
| ***3) Cognitive Disorganisation*** | 0.36 | 0.00 | 1 | - |
| ***4) Parent-rated Negative Symptoms*** | 0.12 | 0.19 | 0.22 | 1 |
|  |  |  |  |  |
| ***TEDS*** |  |  |  |  |
|  | **1** | **2** | **3** | **4** |
| ***1) Paranoia and Hallucinations*** | 1 | - | - | - |
| ***2) Anhedonia*** | 0.05 | 1 | - | - |
| ***3) Cognitive Disorganisation*** | 0.43 | 0.00 | 1 | - |
| ***4) Parent-rated Negative Symptoms*** | 0.15 | 0.17 | 0.21 | 1 |
|  |  |  |  |  |
| ***ALSPAC*** |  |  |  |  |
|  | **1** | **2** | **3** | **4** |
| ***1) Paranoia and Hallucinations*** | 1 | - | - | - |
| ***2) Anhedonia*** | 0.32 | 1 | - | - |
| ***3) Cognitive Disorganisation*** | NA | NA | NA | - |
| ***4) Parent-rated Negative Symptoms*** | 0.09 | 0.22 | NA | 1 |
|  |  |  |  |  |
| ***CATSS*** |  |  |  |  |
|  | **1** | **2** | **3** | **4** |
| ***1) Paranoia and Hallucinations*** | 1 | - | - | - |
| ***2) Anhedonia*** | NA | NA | - | - |
| ***3) Cognitive Disorganisation*** | 0.25 | NA | 1 | - |
| ***4) Parent-rated Negative Symptoms*** | 0.11 | NA | 0.23 | 1 |

Note. Sum scores within each sample were standardized before calculating across sample correlations.

Supplementary Table 7. Pearson’s correlation between raw sum scores and scores after inverse-rank based normalization splitting ties randomly.

| **Sample** | **Psychotic-like Experience** | **Correlation** |
| --- | --- | --- |
| TEDS | Paranoia and Hallucinations | 0.887 |
| TEDS | Anhedonia | 0.990 |
| TEDS | Cognitive Disorganization | 0.971 |
| TEDS | Parent-rated Negative Symptoms | 0.858 |
|  |  |  |
| ALSPAC | Paranoia and Hallucinations | 0.771 |
| ALSPAC | Anhedonia | 0.955 |
| ALSPAC | Parent-rated Negative Symptoms | 0.979 |
|  |  |  |
| CATSS | Paranoia and Hallucinations | 0.723 |
| CATSS | Cognitive Disorganization | 0.992 |
| CATSS | Parent-rated Negative Symptoms | 0.810 |

Supplementary Table 8. Parameters used in AVENGEME analysis.

| **Psych^a^** | ***N*_1_^b^** | **Samp^c^** | **Prev^d^** | **SNP-*h*^2^_1_^e^** | **nSNP^f^** | **Psychotic-like Experience subscale** | ***N*_2_^g^** |
| --- | --- | --- | --- | --- | --- | --- | --- |
| SCZ | 77096 | 0.447 | 0.01 | 0.2618 | 102323 | Paranoia and Hallucinations | 7970 |
| SCZ | 77096 | 0.447 | 0.01 | 0.2618 | 102323 | Paranoia and Hallucinations Excl. zero-scorers | 3845 |
| SCZ | 77096 | 0.447 | 0.01 | 0.2618 | 102323 | Anhedonia | 6068 |
| SCZ | 77096 | 0.447 | 0.01 | 0.2618 | 102323 | Cognitive Disorganization | 5083 |
| SCZ | 77096 | 0.447 | 0.01 | 0.2618 | 102323 | Parent-rated Negative Symptoms | 8763 |
|  |  |  |  |  |  |  |  |
| BD | 16731 | 0.443 | 0.01 | 0.2548 | 67299 | Paranoia and Hallucinations | 7970 |
| BD | 16731 | 0.443 | 0.01 | 0.2548 | 67299 | Anhedonia | 6068 |
| BD | 16731 | 0.443 | 0.01 | 0.2548 | 67299 | Cognitive Disorganization | 5083 |
| BD | 16731 | 0.443 | 0.01 | 0.2548 | 67299 | Parent-rated Negative Symptoms | 8763 |
|  |  |  |  |  |  |  |  |
| MDD | 18759 | 0.493 | 0.15 | 0.1919 | 63107 | Paranoia and Hallucinations | 7970 |
| MDD | 18759 | 0.493 | 0.15 | 0.1919 | 63107 | Anhedonia | 6068 |
| MDD | 18759 | 0.493 | 0.15 | 0.1919 | 63107 | Cognitive Disorganization | 5083 |
| MDD | 18759 | 0.493 | 0.15 | 0.1919 | 63107 | Parent-rated Negative Symptoms | 8763 |

^a^ Psychiatric disorder.

^b^ Number of individuals in training sample.

^c^ Proportion of cases in training sample.

^d^ Prevalence of disorder in general population.

^e^ LDSC estimate of SNP-heritability on a liability scale in training sample.

^f^ Number of LD independent SNPs overlapping between discovery and target samples.

^g^ Effective sample size of target sample.

**Supplementary Table 9.** Mean sex differences for untransformed psychotic-like experience sum scores.

| ***TEDS*** |  |  |  |  |  |
| --- | --- | --- | --- | --- | --- |
|  | **Males** | | **Females** | | *p* |
|  | *µ* | SD | *µ* | SD |  |
| **Paranoia and Hallucinations** | 0.408 | 0.497 | 0.441 | 0.527 | 0.081 |
| **Anhedonia** | 3.630 | 1.544 | 2.866 | 1.459 | 4.85E-41 |
| **Cognitive Disorganisation** | 3.237 | 2.622 | 4.254 | 2.888 | 2.78E-23 |
| **Parent-rated Negative Symptoms** | 0.990 | 1.246 | 0.794 | 1.134 | 1.11E-05 |
|  |  |  |  |  |  |
| ***ALSPAC*** |  |  |  |  |  |
|  | **Males** | | **Females** | | *p* |
|  | *µ* | SD | *µ* | SD |  |
| **Paranoia and Hallucinations** | 0.180 | 0.418 | 0.327 | 0.559 | 3.34E-19 |
| **Anhedonia** | 1.259 | 1.116 | 1.537 | 1.218 | 1.87E-12 |
| **Parent-rated Negative Symptoms** | 1.681 | 1.122 | 1.467 | 1.083 | 7.77E-10 |
|  |  |  |  |  |  |
| ***CATSS*** |  |  |  |  |  |
|  | **Males** | | **Females** | | *p* |
|  | *µ* | SD | *µ* | SD |  |
| **Paranoia and Hallucinations** | 0.154 | 0.381 | 0.233 | 0.461 | 1.94E-05 |
| **Cognitive Disorganization** | 1.824 | 0.939 | 2.048 | 0.998 | 4.24E-11 |
| **Parent-rated Negative Symptoms** | 0.628 | 0.981 | 0.525 | 0.909 | 2.64E-03 |

Note. Mean difference *p*-values were estimated using the two sample t-test.

Supplementary Table 10. Pearson correlation between untransformed psychotic-like experience sum scores and age.

|  | **TEDS** | **ALSPAC** | **CATSS** |
| --- | --- | --- | --- |
| **Paranoia and Hallucinations** | 0.030 | 0.004 | -0.038 |
| **Anhedonia** | -0.018 | -0.025 | NA |
| **Cognitive Disorganisation** | 0.023 | NA | 0.020 |
| **Parent-rated Negative Symptoms** | -0.043 | 0.001 | -0.042 |

Supplementary Table 11. Mega-SNP-heritability estimates from GREML and LD-score regression.

|  | **mega-GREML^a^** | | | **mega-LDSC^b^** | | |
| --- | --- | --- | --- | --- | --- | --- |
|  | **SNP-*h*^2 c^** | **SE** | ***p*** | **SNP-*h*^2 c^** | **SE** | ***p*** |
| Paranoia and Hallucinations | 1.00E-06 | 0.030 | 0.500 | -8.20E-03 | 0.039 | 0.417 |
| Anhedonia | 0.033 | 0.039 | 0.198 | 0.096 | 0.053 | 0.036 |
| Cognitive Disorganisation | 0.050 | 0.046 | 0.138 | 0.136 | 0.068 | 0.022 |
| Parent-rated Negative Symptoms | 1.00E-06 | 0.026 | 0.500 | -0.028 | 0.035 | 0.216 |

^a^ mega-genomic-relatedness-matrix restricted maximum likelihood.

^b^ mega-linkage disequilibrium-score regression.

^c^ Phenotypic variance explained by tagged common genetic variation. ‘SNP’ typically refers to a genetic variant called a single nucleotide polymorphism. However, in the context of SNP-heritability, ‘SNP’ represents all common genetic variation that is tagged using a genome-wide genotyping chip and imputation.

Supplementary Table 12. Independent loci achieving suggestive significance (p < 1x10^-5^) in mega-genome-wide association study of psychotic-like experience domains.

| ***Paranoia and Hallucinations*** | |  |  |  |  |  |  |  |  |
| --- | --- | --- | --- | --- | --- | --- | --- | --- | --- |
| **CHR^a^** | **SNP^b^** | **BP^c^** | **A1^d^** | **A2^e^** | **MAF^f^** | ***β*** | **SE** | ***p*** | **Nearest Gene** |
| 3 | rs73135634 | 84961810 | C | T | 0.202 | -0.093 | 0.019 | 1.33E-06 | *LOC105377193* |
| 17 | rs1008621 | 70362731 | A | C | 0.309 | 0.077 | 0.017 | 4.39E-06 | *LOC146795* |
|  |  |  |  |  |  |  |  |  |  |
| ***Anhedonia*** | |  |  |  |  |  |  |  |  |
| **CHR^a^** | **SNP^b^** | **BP^c^** | **A1^d^** | **A2^e^** | **MAF^f^** | ***β*** | **SE** | ***p*** | **Nearest Gene** |
| 8 | rs149957215 | 39872495 | A | C | 0.013 | -0.417 | 0.076 | 3.76E-08 | *IDO2* |
| 13 | rs78013746 | 61682703 | A | C | 0.027 | 0.255 | 0.054 | 2.37E-06 | *MIR3169* |
| 6 | rs200488 | 27795109 | T | C | 0.018 | 0.297 | 0.063 | 2.89E-06 | *HIST1H4K* |
| 11 | rs117907077 | 11033989 | A | G | 0.024 | -0.286 | 0.062 | 3.27E-06 | *ZBED5−AS1* |
| 6 | rs2531815 | 28436060 | T | C | 0.287 | 0.092 | 0.020 | 4.36E-06 | *ZSCAN23* |
| 20 | rs6033026 | 11059873 | G | A | 0.240 | -0.095 | 0.021 | 5.39E-06 | *LOC339593* |
| 15 | rs7164838 | 34967574 | A | G | 0.317 | 0.088 | 0.019 | 5.55E-06 | *GJD2* |
| 11 | rs2169485 | 41079587 | G | A | 0.178 | -0.109 | 0.024 | 6.02E-06 | *LRRC4C* |
| 10 | rs11195810 | 113835240 | A | G | 0.017 | 0.297 | 0.066 | 6.72E-06 | *GPAM* |
| 14 | rs12897386 | 72471862 | C | T | 0.256 | 0.093 | 0.021 | 7.62E-06 | *RGS6* |
| 9 | rs62545506 | 73241253 | T | G | 0.104 | -0.132 | 0.030 | 8.15E-06 | *TRPM3* |
| 14 | rs34420225 | 94290014 | A | C | 0.212 | -0.100 | 0.022 | 9.38E-06 | *PRIMA1* |
| 15 | rs74519172 | 55010305 | T | C | 0.065 | 0.159 | 0.036 | 9.76E-06 | *UNC13C* |
|  |  |  |  |  |  |  |  |  |  |
| ***Cognitive Disorganization*** | |  |  |  |  |  |  |  |  |
| **CHR^a^** | **SNP^b^** | **BP^c^** | **A1^d^** | **A2^e^** | **MAF^f^** | ***β*** | **SE** | ***p*** | **Nearest Gene** |
| 13 | rs1961120 | 28833372 | C | G | 0.386 | -0.097 | 0.020 | 1.35E-06 | *PAN3* |
| 2 | rs200022365 | 186855226 | T | TTTA | 0.432 | 0.098 | 0.020 | 1.58E-06 | *LOC101927217* |
| 14 | rs7147064 | 47560742 | C | A | 0.474 | -0.092 | 0.019 | 2.41E-06 | *MDGA2* |
| 2 | rs7588854 | 80339218 | A | G | 0.169 | 0.122 | 0.026 | 3.62E-06 | *CTNNA2* |
| 2 | rs80033666 | 170682319 | C | T | 0.089 | -0.154 | 0.033 | 3.80E-06 | *METTL5* |
| 4 | rs1506348 | 126450002 | A | G | 0.060 | -0.184 | 0.040 | 4.70E-06 | *FAT4* |
| 3 | rs185642755 | 85127281 | C | T | 0.012 | -0.358 | 0.079 | 6.37E-06 | *CADM2* |
| 1 | rs6665300 | 65429558 | C | T | 0.014 | 0.368 | 0.083 | 9.66E-06 | *JAK1* |
|  |  |  |  |  |  |  |  |  |  |
| ***Parent-rated Negative Symptoms*** | |  |  |  |  |  |  |  |  |
| **CHR^a^** | **SNP^b^** | **BP^c^** | **A1^d^** | **A2^e^** | **MAF^f^** | ***β*** | **SE** | ***p*** | **Nearest Gene** |
| 5 | rs147205145 | 36033829 | A | G | 0.011 | 0.366 | 0.071 | 2.69E-07 | *UGT3A2* |
| 4 | rs4400001 | 38212771 | A | C | 0.391 | 0.069 | 0.016 | 8.56E-06 | *LOC105374408* |
| 8 | rs72334712 | 108862133 | CT | C | 0.046 | 0.158 | 0.036 | 9.09E-06 | *RSPO2* |
| 8 | rs35428606 | 101649797 | T | C | 0.084 | -0.114 | 0.026 | 9.25E-06 | *SNX31* |
| 7 | rs62457829 | 29549919 | C | G | 0.082 | 0.122 | 0.028 | 9.80E-06 | *CHN2* |

^a^ Chromosome number.

^b^ Single nucleotide polymorphism.

^c^ Base-pair position.

^d^ Allele 1.

^e^ Allele 2.

^f^ Minor allele frequency.

Supplementary Table 13. Top ten genes associated with psychotic-like experience domains using MAGMA.

| ***Paranoia and Hallucinations*** | | |  |  |  |
| --- | --- | --- | --- | --- | --- |
| **CHR^a^** | **Gene Symbol** | **NSNPS^b^** | ***z*** | ***p*** | **Corrected *p^c^*** |
| 4 | *GLRB* | 215 | 3.816 | 6.79E-05 | 1.000 |
| 2 | *UGGT1* | 312 | 3.708 | 1.04E-04 | 1.000 |
| 14 | *ACYP1* | 46 | 3.694 | 1.10E-04 | 1.000 |
| 14 | *NEK9* | 88 | 3.690 | 1.12E-04 | 1.000 |
| 2 | *PDK1* | 263 | 3.689 | 1.12E-04 | 1.000 |
| 12 | *EMP1* | 43 | 3.671 | 1.21E-04 | 1.000 |
| 14 | *MLH3* | 63 | 3.632 | 1.41E-04 | 1.000 |
| 2 | *GPC1* | 41 | 3.631 | 1.41E-04 | 1.000 |
| 14 | *ZC2HC1C* | 41 | 3.631 | 1.41E-04 | 1.000 |
| 14 | *TMED10* | 85 | 3.627 | 1.43E-04 | 1.000 |
|  |  |  |  |  |  |
| ***Anhedonia*** | |  |  |  |  |
| **CHR^a^** | **Gene Symbol** | **NSNPS^b^** | ***z*** | ***p*** | **Corrected *p^c^*** |
| 14 | *ABHD12B* | 36 | 4.045 | 2.62E-05 | 0.451 |
| 15 | *MAPKBP1* | 91 | 3.957 | 3.79E-05 | 0.653 |
| 10 | *BNIP3* | 37 | 3.657 | 1.27E-04 | 1.000 |
| 22 | *CECR5* | 43 | 3.634 | 1.40E-04 | 1.000 |
| 14 | *PYGL* | 126 | 3.615 | 1.50E-04 | 1.000 |
| 10 | *PPP2R2D* | 73 | 3.607 | 1.55E-04 | 1.000 |
| 15 | *C15orf27* | 199 | 3.592 | 1.64E-04 | 1.000 |
| 12 | *IGFBP6* | 40 | 3.588 | 1.66E-04 | 1.000 |
| 19 | *ZNF43* | 143 | 3.551 | 1.92E-04 | 1.000 |
| 15 | *JMJD7* | 76 | 3.530 | 2.08E-04 | 1.000 |
|  |  |  |  |  |  |
| ***Cognitive Disorganization*** | | |  |  |  |
| **CHR^a^** | **Gene Symbol** | **NSNPS^b^** | ***z*** | ***p*** | **Corrected *p^c^*** |
| 13 | *CLYBL* | 482 | 4.362 | 6.45E-06 | 0.111 |
| 19 | *PSG4* | 1 | 3.969 | 3.61E-05 | 0.621 |
| 12 | *ASUN* | 100 | 3.961 | 3.74E-05 | 0.644 |
| 4 | *RUFY3* | 248 | 3.817 | 6.77E-05 | 1.000 |
| 9 | *ANP32B* | 52 | 3.642 | 1.35E-04 | 1.000 |
| 7 | *PEX1* | 57 | 3.617 | 1.49E-04 | 1.000 |
| 7 | *GATAD1* | 40 | 3.477 | 2.54E-04 | 1.000 |
| 9 | *NR5A1* | 8 | 3.447 | 2.84E-04 | 1.000 |
| 7 | *ERVW-1* | 46 | 3.436 | 2.95E-04 | 1.000 |
| 4 | *MOB1B* | 93 | 3.388 | 3.53E-04 | 1.000 |
|  |  |  |  |  |  |
| ***Parent-rated Negative Symptoms*** | | | |  |  |
| **CHR^a^** | **Gene Symbol** | **NSNPS^b^** | ***z*** | ***p*** | **Corrected *p^c^*** |
| 20 | *SDCBP2* | 30 | 3.964 | 3.69E-05 | 0.636 |
| 2 | *TRAPPC12* | 169 | 3.888 | 5.06E-05 | 0.872 |
| 2 | *CD28* | 76 | 3.861 | 5.65E-05 | 0.973 |
| 11 | *FUT4* | 23 | 3.852 | 5.87E-05 | 1.000 |
| 5 | *SPEF2* | 594 | 3.790 | 7.54E-05 | 1.000 |
| 1 | *SPAG17* | 351 | 3.750 | 8.83E-05 | 1.000 |
| 4 | *GC* | 115 | 3.669 | 1.22E-04 | 1.000 |
| 11 | *GPR83* | 157 | 3.538 | 2.02E-04 | 1.000 |
| 8 | *DPY19L4* | 103 | 3.421 | 3.12E-04 | 1.000 |
| 4 | *NPY2R* | 175 | 3.393 | 3.45E-04 | 1.000 |

^a^ Chromosome number.

^b^ Number of SNPs.

^c^ Bonferroni corrected *p-*value.

Supplementary Table 14. Top ten differentially-expressed genes for psychotic-like experience domains based on predicted gene expression levels.

| ***Paranoia and Hallucinations*** | | |  |  |
| --- | --- | --- | --- | --- |
| **Gene Symbol** | **BETA^a^** | **SE** | ***p*** | **Corrected *p^b^*** |
| *NAGPA* | -1.389 | 0.411 | 7.32E-04 | 1.000 |
| *OTUD3* | 0.164 | 0.050 | 9.21E-04 | 1.000 |
| *POLR2J* | 1.113 | 0.343 | 1.19E-03 | 1.000 |
| *SWI5* | 0.262 | 0.083 | 1.53E-03 | 1.000 |
| *COMMD2* | 0.230 | 0.073 | 1.73E-03 | 1.000 |
| *C6orf106* | 0.138 | 0.045 | 2.16E-03 | 1.000 |
| *SLX4* | -0.275 | 0.090 | 2.24E-03 | 1.000 |
| *PLPPR2* | -0.311 | 0.104 | 2.84E-03 | 1.000 |
| *SMARCC2* | -0.414 | 0.142 | 3.64E-03 | 1.000 |
| *NCLN* | -1.248 | 0.439 | 4.49E-03 | 1.000 |
|  |  |  |  |  |
| ***Anhedonia*** |  |  |  |  |
| **Gene Symbol** | **BETA** | **SE** | ***p*** | **Corrected *p*** |
| *CIB2* | -1.207 | 0.317 | 1.42E-04 | 0.392 |
| *INTS1* | -0.192 | 0.054 | 3.58E-04 | 0.991 |
| *FAM198A* | 0.247 | 0.073 | 7.63E-04 | 1.000 |
| *ACKR2* | -0.483 | 0.146 | 9.69E-04 | 1.000 |
| *STRN* | -2.089 | 0.641 | 1.11E-03 | 1.000 |
| *AGO2* | 0.252 | 0.082 | 2.16E-03 | 1.000 |
| *TEAD2* | -0.258 | 0.087 | 3.07E-03 | 1.000 |
| *CBLN4* | -0.715 | 0.248 | 3.94E-03 | 1.000 |
| *NA* | 0.643 | 0.225 | 4.34E-03 | 1.000 |
| *ZNF514* | -0.243 | 0.086 | 4.47E-03 | 1.000 |
|  |  |  |  |  |
| ***Cognitive Disorganization*** | | |  |  |
| **Gene Symbol** | **BETA** | **SE** | ***p*** | **Corrected *p*** |
| *HACD2* | -0.153 | 0.030 | 2.47E-07 | 6.83E-04 |
| *RASAL2* | 0.200 | 0.055 | 2.58E-04 | 0.715 |
| *AP4S1* | -0.162 | 0.049 | 9.18E-04 | 1.000 |
| *LRBA* | -0.419 | 0.129 | 1.13E-03 | 1.000 |
| *BRIX1* | -0.293 | 0.092 | 1.47E-03 | 1.000 |
| *TRMO* | -0.174 | 0.055 | 1.54E-03 | 1.000 |
| *RAB43* | 0.219 | 0.069 | 1.61E-03 | 1.000 |
| *NOL6* | 0.339 | 0.109 | 1.87E-03 | 1.000 |
| *C2orf88* | 0.169 | 0.055 | 2.30E-03 | 1.000 |
| *SPON2* | -1.841 | 0.608 | 2.45E-03 | 1.000 |
|  |  |  |  |  |
| ***Parent-rated Negative Symptoms*** | | | |  |
| **Gene Symbol** | **BETA** | **SE** | ***p*** | **Corrected *p*** |
| *STXBP5L* | -0.148 | 0.040 | 1.89E-04 | 0.523 |
| *AKAP3* | -0.121 | 0.033 | 2.59E-04 | 0.718 |
| *VPS50* | -0.360 | 0.118 | 2.28E-03 | 1.000 |
| *DR1* | 0.208 | 0.068 | 2.32E-03 | 1.000 |
| *PDCD5* | -0.144 | 0.047 | 2.43E-03 | 1.000 |
| *LILRA6* | -0.365 | 0.122 | 2.79E-03 | 1.000 |
| *SELL* | 0.411 | 0.138 | 2.85E-03 | 1.000 |
| *CTC-236F12.4* | -0.553 | 0.188 | 3.22E-03 | 1.000 |
| *SULT4A1* | 0.256 | 0.089 | 3.87E-03 | 1.000 |
| *ADSS* | -0.156 | 0.054 | 3.93E-03 | 1.000 |

^a^ Unstandardized effect size.

^b^ Bonferroni corrected *p*-value.

Supplementary Table 15. Schizophrenia polygenic risk score predicting psychotic-like experience domains at 8 *p*-value thresholds.

| ***Paranoia and Hallucinations*** | | |  |  |
| --- | --- | --- | --- | --- |
| ***p*T^a^** | ***β*** | **SE** | ***p*** | ***r*^2^** |
| 0.001 | 0.002 | 0.011 | 0.889 | 0.000% |
| 0.01 | -0.001 | 0.011 | 0.926 | 0.000% |
| 0.05 | 0.001 | 0.011 | 0.915 | 0.000% |
| 0.1 | -0.002 | 0.011 | 0.824 | 0.001% |
| 0.2 | -0.005 | 0.011 | 0.664 | 0.002% |
| 0.3 | -0.002 | 0.011 | 0.848 | 0.000% |
| 0.4 | -0.002 | 0.011 | 0.832 | 0.001% |
| 0.5 | -0.002 | 0.011 | 0.892 | 0.000% |
| ***Paranoia and Hallucinations (excl. zero-scorers)*** | | | |  |
| ***p*T^a^** | ***β*** | **SE** | ***p*** | ***r*^2^** |
| 0.001 | 0.031 | 0.012 | 0.008 | 0.094% |
| 0.01 | 0.026 | 0.012 | 0.025 | 0.067% |
| 0.05 | 0.019 | 0.012 | 0.098 | 0.037% |
| 0.1 | 0.017 | 0.012 | 0.156 | 0.028% |
| 0.3 | 0.015 | 0.012 | 0.208 | 0.022% |
| 0.4 | 0.012 | 0.012 | 0.311 | 0.014% |
| 0.5 | 0.011 | 0.012 | 0.336 | 0.013% |
| 0.2 | 0.010 | 0.012 | 0.404 | 0.010% |
| ***Anhedonia*** |  |  |  |  |
| ***p*T^a^** | ***β*** | **SE** | ***p*** | ***r*^2^** |
| 0.001 | 0.021 | 0.013 | 0.104 | 0.043% |
| 0.01 | 0.025 | 0.013 | 0.050 | 0.063% |
| 0.05 | 0.022 | 0.013 | 0.083 | 0.050% |
| 0.1 | 0.028 | 0.013 | 0.030 | 0.079% |
| 0.2 | 0.028 | 0.013 | 0.034 | 0.076% |
| 0.3 | 0.023 | 0.013 | 0.077 | 0.052% |
| 0.4 | 0.024 | 0.013 | 0.059 | 0.059% |
| 0.5 | 0.027 | 0.013 | 0.039 | 0.071% |
| ***Cognitive Disorganisation*** | | |  |  |
| ***p*T^a^** | ***β*** | **SE** | ***p*** | ***r*^2^** |
| 0.001 | 0.024 | 0.014 | 0.081 | 0.057% |
| 0.01 | 0.029 | 0.014 | 0.035 | 0.083% |
| 0.05 | 0.028 | 0.014 | 0.048 | 0.076% |
| 0.1 | 0.026 | 0.014 | 0.065 | 0.067% |
| 0.2 | 0.024 | 0.014 | 0.082 | 0.059% |
| 0.3 | 0.028 | 0.014 | 0.048 | 0.077% |
| 0.4 | 0.027 | 0.014 | 0.056 | 0.072% |
| 0.5 | 0.028 | 0.014 | 0.050 | 0.076% |
| ***Negative Symptoms*** | |  |  |  |
| ***p*T^a^** | ***β*** | **SE** | ***p*** | ***r*^2^** |
| 0.001 | 0.019 | 0.011 | 0.078 | 0.034% |
| 0.01 | 0.026 | 0.011 | 0.015 | 0.067% |
| 0.05 | 0.030 | 0.011 | 0.005 | 0.088% |
| 0.1 | 0.025 | 0.011 | 0.021 | 0.061% |
| 0.2 | 0.026 | 0.011 | 0.014 | 0.069% |
| 0.3 | 0.027 | 0.011 | 0.012 | 0.073% |
| 0.4 | 0.024 | 0.011 | 0.023 | 0.059% |
| 0.5 | 0.024 | 0.011 | 0.027 | 0.057% |

^a^ *p*-value threshold used to select genetic variation included in risk score calculation.

Supplementary Table 16. Bipolar disorder polygenic risk score predicting psychotic-like experience domains at 8 *p*-value thresholds.

| ***Paranoia and Hallucinations*** | | |  |  |
| --- | --- | --- | --- | --- |
| ***p*T** | ***β*** | **SE** | ***p*** | ***r*^2^** |
| 0.001 | -0.022 | 0.011 | 0.051 | 0.049% |
| 0.010 | -0.034 | 0.011 | 0.002 | 0.115% |
| 0.050 | -0.028 | 0.011 | 0.014 | 0.076% |
| 0.100 | -0.026 | 0.011 | 0.019 | 0.069% |
| 0.200 | -0.021 | 0.011 | 0.062 | 0.043% |
| 0.300 | -0.019 | 0.011 | 0.095 | 0.035% |
| 0.400 | -0.019 | 0.011 | 0.094 | 0.035% |
| 0.500 | -0.020 | 0.011 | 0.077 | 0.039% |
|  |  |  |  |  |
| ***Anhedonia*** |  |  |  |  |
| ***p*T** | ***β*** | **SE** | ***p*** | ***r*^2^** |
| 0.001 | 0.007 | 0.013 | 0.600 | 0.004% |
| 0.010 | 0.010 | 0.013 | 0.448 | 0.009% |
| 0.050 | -0.013 | 0.013 | 0.304 | 0.017% |
| 0.100 | -0.017 | 0.013 | 0.178 | 0.030% |
| 0.200 | -0.011 | 0.013 | 0.385 | 0.013% |
| 0.300 | -0.014 | 0.013 | 0.269 | 0.021% |
| 0.400 | -0.011 | 0.013 | 0.391 | 0.012% |
| 0.500 | -0.011 | 0.013 | 0.399 | 0.012% |
|  |  |  |  |  |
| ***Cognitive Disorganization*** | | |  |  |
| ***p*T** | ***β*** | **SE** | ***p*** | ***r*^2^** |
| 0.001 | -0.013 | 0.014 | 0.333 | 0.017% |
| 0.010 | -0.001 | 0.014 | 0.937 | 0.000% |
| 0.050 | 0.008 | 0.013 | 0.541 | 0.007% |
| 0.100 | 0.005 | 0.013 | 0.725 | 0.002% |
| 0.200 | -0.001 | 0.013 | 0.964 | 0.000% |
| 0.300 | 0.006 | 0.013 | 0.665 | 0.003% |
| 0.400 | 0.006 | 0.013 | 0.670 | 0.003% |
| 0.500 | 0.004 | 0.013 | 0.754 | 0.002% |
|  |  |  |  |  |
| ***Parent-rated Negative Symptoms*** | | |  |  |
| ***p*T** | ***β*** | **SE** | ***p*** | ***r*^2^** |
| 0.001 | -0.009 | 0.011 | 0.425 | 0.007% |
| 0.010 | 0.002 | 0.011 | 0.837 | 0.000% |
| 0.050 | 0.003 | 0.011 | 0.786 | 0.001% |
| 0.100 | -0.002 | 0.011 | 0.858 | 0.000% |
| 0.200 | -0.005 | 0.011 | 0.626 | 0.003% |
| 0.300 | -0.007 | 0.011 | 0.506 | 0.005% |
| 0.400 | -0.006 | 0.011 | 0.551 | 0.004% |
| 0.500 | -0.009 | 0.011 | 0.388 | 0.008% |

^a^ *p*-value threshold used to select genetic variation included in risk score calculation.

Supplementary Table 17. Major depression polygenic risk score predicting psychotic-like experience domains at 8 *p*-value thresholds.

| ***Paranoia and Hallucinations*** | | |  |  |
| --- | --- | --- | --- | --- |
| ***p*T** | ***β*** | **SE** | ***p*** | ***r*^2^** |
| 0.001 | 0.002 | 0.011 | 0.861 | 0.000% |
| 0.010 | -0.002 | 0.011 | 0.835 | 0.001% |
| 0.050 | 0.002 | 0.011 | 0.877 | 0.000% |
| 0.100 | 0.006 | 0.011 | 0.589 | 0.004% |
| 0.200 | -0.002 | 0.011 | 0.892 | 0.000% |
| 0.300 | 0.004 | 0.011 | 0.687 | 0.002% |
| 0.400 | 0.000 | 0.011 | 0.980 | 0.000% |
| 0.500 | 0.001 | 0.011 | 0.926 | 0.000% |
|  |  |  |  |  |
| ***Anhedonia*** |  |  |  |  |
| ***p*T** | ***β*** | **SE** | ***p*** | ***r*^2^** |
| 0.001 | 0.003 | 0.013 | 0.794 | 0.001% |
| 0.010 | 0.019 | 0.013 | 0.135 | 0.037% |
| 0.050 | 0.022 | 0.013 | 0.083 | 0.048% |
| 0.100 | 0.023 | 0.013 | 0.069 | 0.053% |
| 0.200 | 0.024 | 0.013 | 0.062 | 0.057% |
| 0.300 | 0.029 | 0.013 | 0.025 | 0.082% |
| 0.400 | 0.032 | 0.013 | 0.014 | 0.100% |
| 0.500 | 0.033 | 0.013 | 0.010 | 0.109% |
|  |  |  |  |  |
| ***Cognitive Disorganization*** | | |  |  |
| ***p*T** | ***β*** | **SE** | ***p*** | ***r*^2^** |
| 0.001 | -0.012 | 0.014 | 0.403 | 0.014% |
| 0.010 | 0.010 | 0.014 | 0.479 | 0.009% |
| 0.050 | 0.018 | 0.014 | 0.189 | 0.033% |
| 0.100 | 0.015 | 0.014 | 0.285 | 0.021% |
| 0.200 | 0.007 | 0.014 | 0.610 | 0.005% |
| 0.300 | 0.014 | 0.014 | 0.316 | 0.019% |
| 0.400 | 0.014 | 0.014 | 0.296 | 0.021% |
| 0.500 | 0.015 | 0.014 | 0.276 | 0.022% |
|  |  |  |  |  |
| ***Parent-rated Negative Symptoms*** | | |  |  |
| ***p*T** | ***β*** | **SE** | ***p*** | ***r*^2^** |
| 0.001 | 0.028 | 0.011 | 0.008 | 0.078% |
| 0.010 | 0.020 | 0.011 | 0.061 | 0.040% |
| 0.050 | 0.021 | 0.011 | 0.051 | 0.044% |
| 0.100 | 0.013 | 0.011 | 0.226 | 0.018% |
| 0.200 | 0.012 | 0.011 | 0.271 | 0.014% |
| 0.300 | 0.014 | 0.011 | 0.187 | 0.021% |
| 0.400 | 0.012 | 0.011 | 0.283 | 0.013% |
| 0.500 | 0.012 | 0.011 | 0.273 | 0.014% |

^a^ *p*-value threshold used to select genetic variation included in risk score calculation.

Supplementary Table 18. Comparison of schizophrenia, bipolar disorder, and major depression polygenic risk scores in low and high psychotic-like experience domain groups. Low and high groups were defined as the bottom and top 25% of raw psychotic-like experience domain sum scores. This table shows results when using polygenic risk scores at the most predictive p-value threshold for each trait. Linear regression results for the same p-value thresholds are shown in Table 3 of the main text.

| **Schizophrenia** |  |  |  |
| --- | --- | --- | --- |
|  | **OR** | **CI 95%^a^** | ***p*** |
| Paranoia and Hallucinations  Excluding zero-scorers | 0.997  1.077 | 0.049  .077 | 0.894  0.059 |
| Anhedonia | 1.073 | 0.067 | **0.039** |
| Cognitive Disorganization | 1.11 | 0.07 | **3.82E-03** |
| Parent-rated Negative Symptoms | 1.084 | 0.059 | **7.42E-03** |
|  |  |  |  |
| **Bipolar Disorder** |  |  |  |
|  | **OR** | **CI 95%^a^** | ***p*** |
| Paranoia and Hallucinations | 0.903 | 0.05 | **5.90E-05** |
| Anhedonia | 0.967 | 0.068 | 0.338 |
| Cognitive Disorganization | 0.999 | 0.071 | 0.968 |
| Parent-rated Negative Symptoms | 0.974 | 0.058 | 0.384 |
|  |  |  |  |
| **Major Depression** |  |  |  |
|  | **OR** | **CI 95%^a^** | ***p*** |
| Paranoia and Hallucinations | 1.032 | 0.05 | 0.218 |
| Anhedonia | 1.102 | 0.067 | **4.59E-03** |
| Cognitive Disorganization | 1.061 | 0.071 | 0.099 |
| Parent-rated Negative Symptoms | 1.062 | 0.058 | **0.042** |

^a^ 95% confidence interval

Supplementary Figure 1. LocusZoom plot of genome-wide significant association with Anhedonia.

Note. chr8 = chromosome 8, Mb = megabase.

Supplementary Figure 2. Manhattan plot of Paranoia and Hallucinations mega-GWAS.


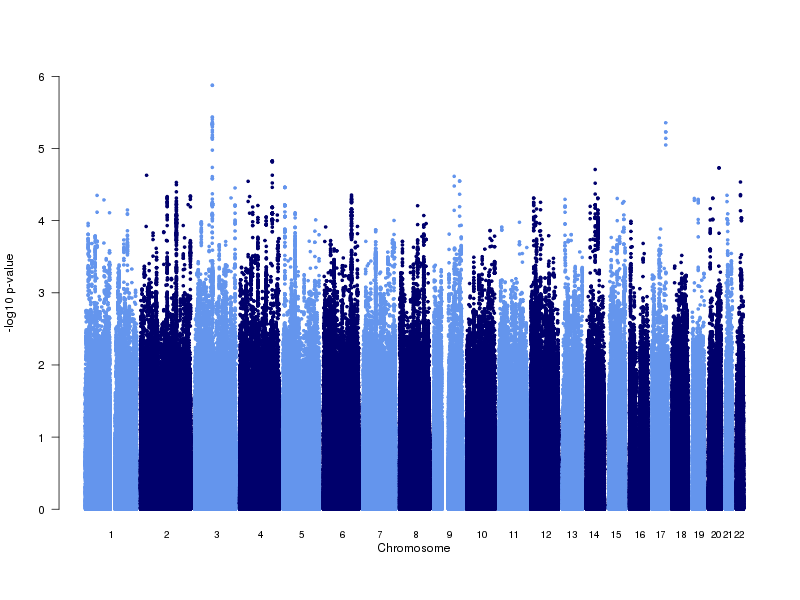


Supplementary Figure 3. Manhattan plot of Anhedonia mega-GWAS.


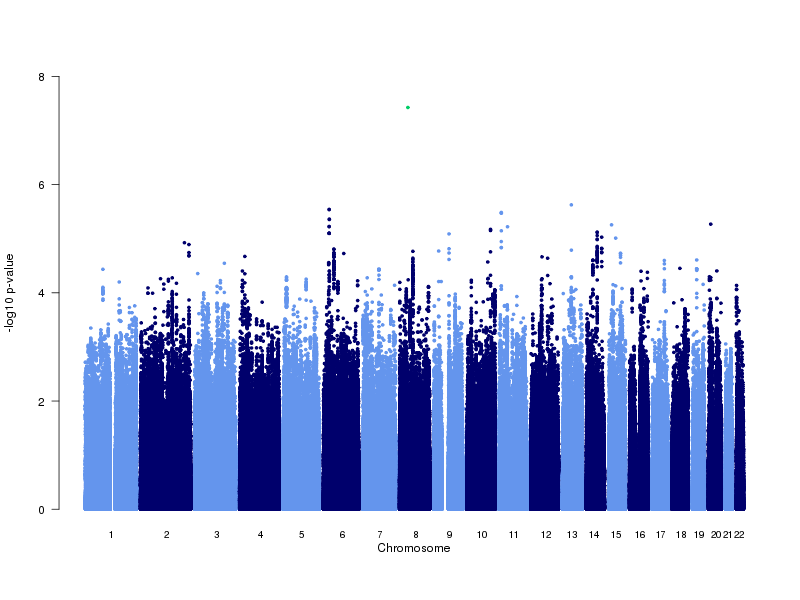


Supplementary Figure 4: Manhattan plot of Cognitive Disorganization mega-GWAS.


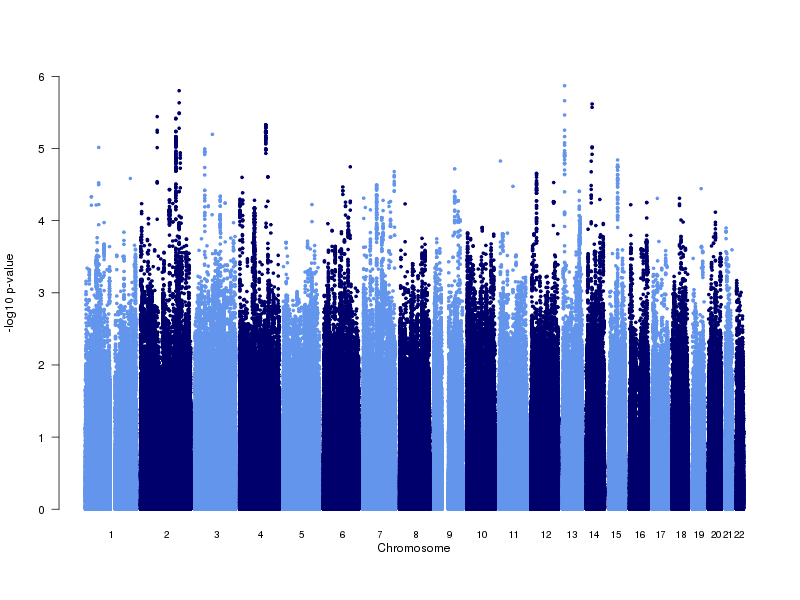


Supplementary Figure 5. Manhattan plot of Parent-rated Negative Symptoms mega-GWAS.


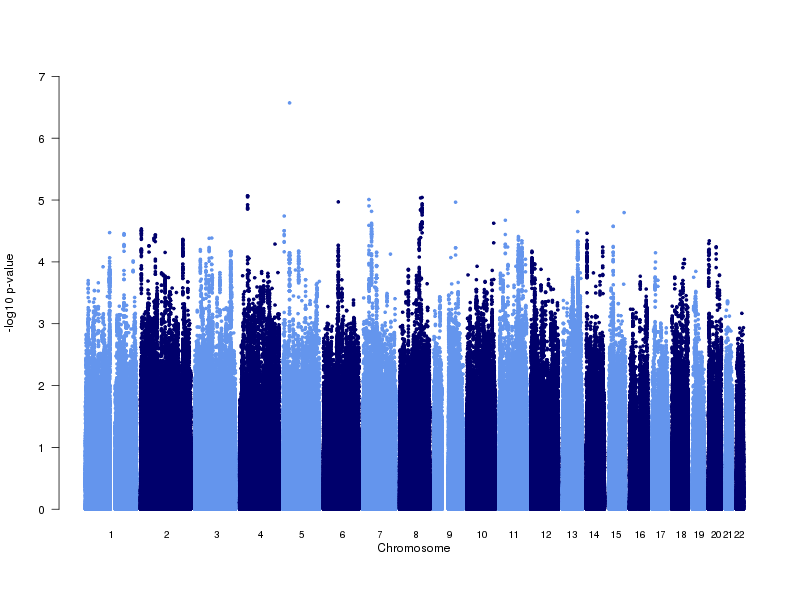


Supplementary Figure 6. Quantile-quantile plot of psychotic-like experience domain mega-GWASs: A) Paranoia and Hallucinations, B) Anhedonia, C) Cognitive Disorganization, D) Parent-rated Negative Symptoms.

Supplementary Figure 7. Schizophrenia polygenic risk score predicting psychotic-like experience domains in adolescence. Linear regression results are shown for polygenic risk scores at all *p*-value thresholds. Figure corresponds to results shown in Supplementary Table 13. Error bars indicate 95% confidence intervals.

Note. * = *p* ≤ 0.05, ** = *p* ≤ 0.01.

Supplementary Figure 8. Bipolar disorder polygenic risk score predicting psychotic-like experience domains in adolescence. Linear regression results are shown for polygenic risk scores at all p-value thresholds. Figure corresponds to results shown in Supplementary Table 14. Error bars indicate 95% confidence intervals.

Note. * = *p* ≤ 0.05, ** = *p* ≤ 0.01.

Supplementary Figure 9. Major depression polygenic risk score predicting psychotic-like experience domains in adolescence. Linear regression results are shown for polygenic risk scores at all p-value thresholds. Figure corresponds to results shown in Supplementary Table 15. Error bars indicate 95% confidence intervals.

Note. * = *p* ≤ 0.05, ** = *p* ≤ 0.01.

Supplementary Figure 10. Schizophrenia, bipolar disorder, and major depression polygenic risk score mean differences between low- and high-scoring psychotic-like experience domain groups. Polygenic risk scores were adjusted to control for covariate effects. Low- and high-scoring groups determined as the bottom and top 25% of raw psychotic-like experience domain sum scores. This plot shows mean differences for polygenic risk scores at the most predictive *p*-value threshold for each trait. Significance of mean difference was determined using logistic regression (results shown in Supplementary Table 16). Error bars indicate the standard error of the mean.

Note. * = *p* ≤ 0.05, ** = *p* ≤ 0.01, *** = *p* ≤ 0.001.

Supplementary Figure 11. Local polynomial regression of schizophrenia polygenic risk score (*p*-value threshold of *p*<0.3) and Paranoia and Hallucinations. The red line indicates the schizophrenia polygenic risk score (left y-axis) of individuals across the Paranoia and Hallucinations distribution. Histogram in background shows number of individuals (N, right y-axis) across the Paranoia and Hallucinations distribution.

**Supplementary references:**

Anckarsäter H, Lundström S, Kollberg L, Kerekes N, Palm C, Carlström E, Långström N, Magnusson PKE, Halldner L, Bölte S. 2011. The child and adolescent twin study in Sweden (CATSS). Twin Res. Hum. Genet. 14: 495–508.

Boyd A, Golding J, Macleod J, Lawlor DA, Fraser A, Henderson J, Molloy L, Ness A, Ring S, Smith GD. 2012. Cohort profile: the “children of the 90s”—the index offspring of the Avon Longitudinal Study of Parents and Children. Int. J. Epidemiol.: dys064.

Docherty SJ, Davis OSP, Kovas Y, Meaburn EL, Dale PS, Petrill SA, Schalkwyk LC, Plomin R. 2010. A genome‐wide association study identifies multiple loci associated with mathematics ability and disability. Genes, Brain Behav. 9: 234–247.

Gamazon ER, Wheeler HE, Shah KP, Mozaffari S V, Aquino-Michaels K, Carroll RJ, Eyler AE, Denny JC, Nicolae DL, Cox NJ. 2015. A gene-based association method for mapping traits using reference transcriptome data. Nat. Genet. 47: 1091–1098.

Haworth CMA, Davis OSP, Plomin R. 2013. Twins Early Development Study (TEDS): a genetically sensitive investigation of cognitive and behavioral development from childhood to young adulthood. Twin Res. Hum. Genet. 16: 117–125.

Howie B, Marchini J, Stephens M. 2011. Genotype imputation with thousands of genomes. G3 Genes, Genomes, Genet. 1: 457–470.

Howie BN, Donnelly P, Marchini J. 2009. A flexible and accurate genotype imputation method for the next generation of genome-wide association studies. PLoS Genet 5: e1000529.

Kelleher I, Harley M, Murtagh A, Cannon M. 2011. Are screening instruments valid for psychotic-like experiences? A validation study of screening questions for psychotic-like experiences using in-depth clinical interview. Schizophr. Bull. 37: 362–369.

De Leeuw CA, Mooij JM, Heskes T, Posthuma D. 2015. MAGMA: generalized gene-set analysis of GWAS data. PLoS Comput Biol 11: e1004219.

Magnusson PKE, Almqvist C, Rahman I, Ganna A, Viktorin A, Walum H, Halldner L, Lundström S, Ullén F, Långström N. 2013. The Swedish Twin Registry: establishment of a biobank and other recent developments. Twin Res. Hum. Genet. 16: 317–329.

Ronald A, Sieradzka D, Cardno AG, Haworth CM a, McGuire P, Freeman D. 2014. Characterization of psychotic experiences in adolescence using the specific psychotic experiences questionnaire: Findings from a study of 5000 16-Year-Old Twins. Schizophr. Bull. 40: 868–877.

Zammit S, Owen MJ, Evans J, Heron J, Lewis G. 2011. Cannabis, COMT and psychotic experiences. Br. J. Psychiatry 199: 380–385.
